# Supplementary material for: Monte Carlo simulations for the evaluation of oligomerization data in TOCCSL experiments
Source: Biophys J. 2023 Apr 23;122(11):2367–80. doi: 10.1016/j.bpj.2023.04.021 (PMC10257147; doi:10.1016/j.bpj.2023.04.021)
Supplement: Document S1. Figures S1–S11 and Tables S1–S7 [file mmc1.pdf]

Biophysical Journal, Volume *122*

Supplemental information

Monte Carlo simulations for the evaluation of oligomerization data in TOCCSL experiments

Clara Bodner, Dominik Kiesenhofer, Gerhard J. Schütz, and Mario Brameshuber

Supplemental information Bodner et al., “*In silico* **TOCCSL experiments**”

Supplemental figures

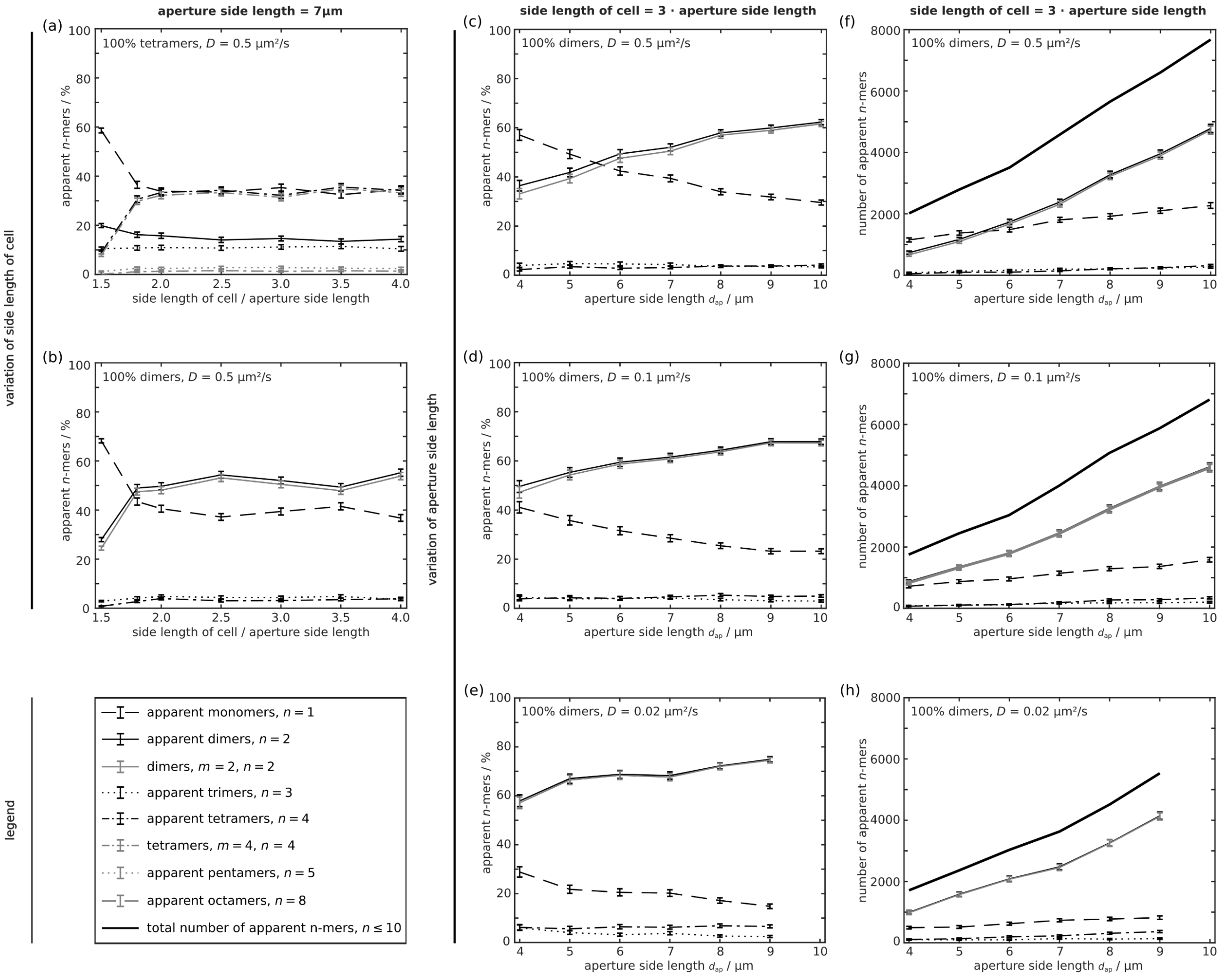

Figure S1 The influence of the simulated cell area (assuming an aperture side length of $d_{ap} = 7$ µm) (a, b) and the influence of the aperture side length d_{ap} (c-h, assuming the side length of the cell to be $3 \cdot d_{ap}$) on the apparent *n*-mer fractions were studied. A purely tetrameric population with $D = 0.5$ µm²/s (a) and purely dimeric populations with $D = 0.5$ µm²/s (b, c, f), $D = 0.1$ µm²/s (d, g) or $D = 0.02$ µm²/s (e, h) were exposed to photobleaching for $t_{bleach} = 4$ s assuming a diffraction-affected laser intensity profile with an intensity decay outside the aperture-restricted region reaching 0 after $d_{edge} = 1$ µm. The optimal t_{rec} and the according analysis region were determined individually for each cell area and each d_{ap}. The detection of apparent monomers (black dashed line) is caused by partial photobleaching, which reduces the fraction of apparent dimers (black solid line). Apparent trimers (dotted line) and apparent tetramers (dashed-dotted line) are present due to random colocalizations. The gray solid line depicts the fraction of dimers excluding apparent dimers due to random colocalizations. Apparent *n*-mer fractions for $n > 4$ are only shown in (a). (a,b): For cell side lengths smaller than 2 times the aperture side length, depletion of fluorescent molecules has to be taken into account. Due to the small cell size and the small, finite reservoir of molecules, most molecules on the cell area are either fully or partially photobleached during illumination. Hence, the probability for the same oligomer to leave and enter the aperture restricted region multiple times during photobleaching is higher compared to larger cell sizes. The high number of partially photobleached oligomers results in a lower fraction of apparent tetramers (a) and apparent dimers (b), compared to larger cell sizes. (c-h): For increasing d_{ap} the recovery time t_{rec} and the size of the analysis region increase and thus, the number as well as the fraction of apparent dimers increase, whereas the fraction of apparent monomers decreases. Shown are the mean apparent *n*-mer fractions (a-e) and the number of apparent *n*-mers (sum over 1000 simulation runs) (f-h) and the error bar (95 % bootstrapping confidence interval) from 1000 independent simulations. The lines are a guide to the eye.

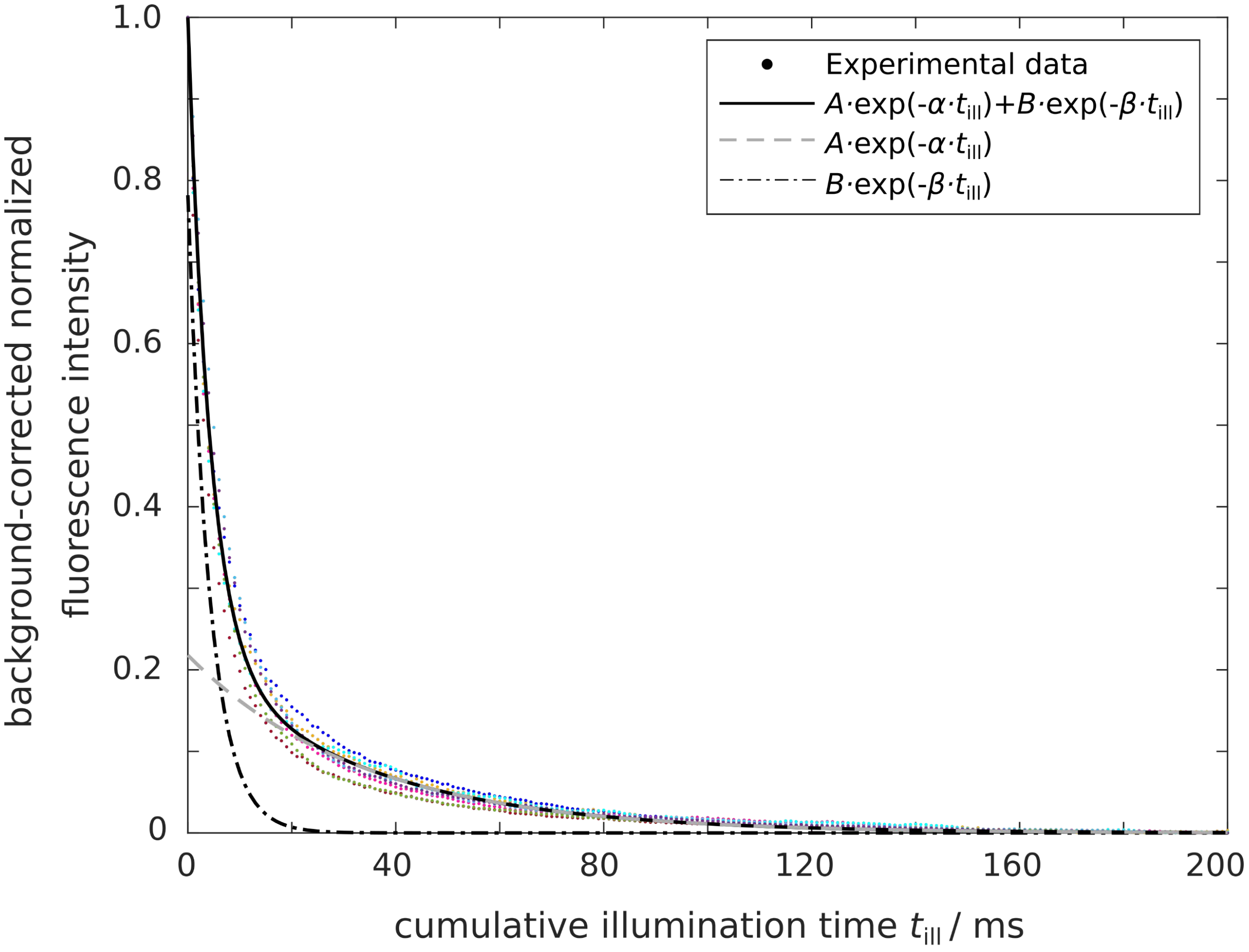

Figure S2 A photobleaching curve was experimentally determined from 8 independent CHO mGFP-GPI cells (colored dots). For each cell 200 images were recorded with an illumination time of $t_{ill} = 1$ ms. Hence, cells were exposed to photobleaching for a time given by the cumulative illumination time of $n \cdot 1\ ms$, with n denoting the frame number. For all 200 images the integrated brightness of the same region of interest (ROI) within the illuminated area was determined, background-corrected, normalized, and fitted via linear least squares by a two-component exponential function. The mean fit parameters derived from 8 cells, $a = 235$ 1/s, $\beta = 30$ 1/s, $A = 0.782$ and $B = 0.218$, were used to calculate the photobleaching curve (black line). For the simulations, one photobleaching rate coefficient α or β is assigned to each dye molecule according to its weight A or B.

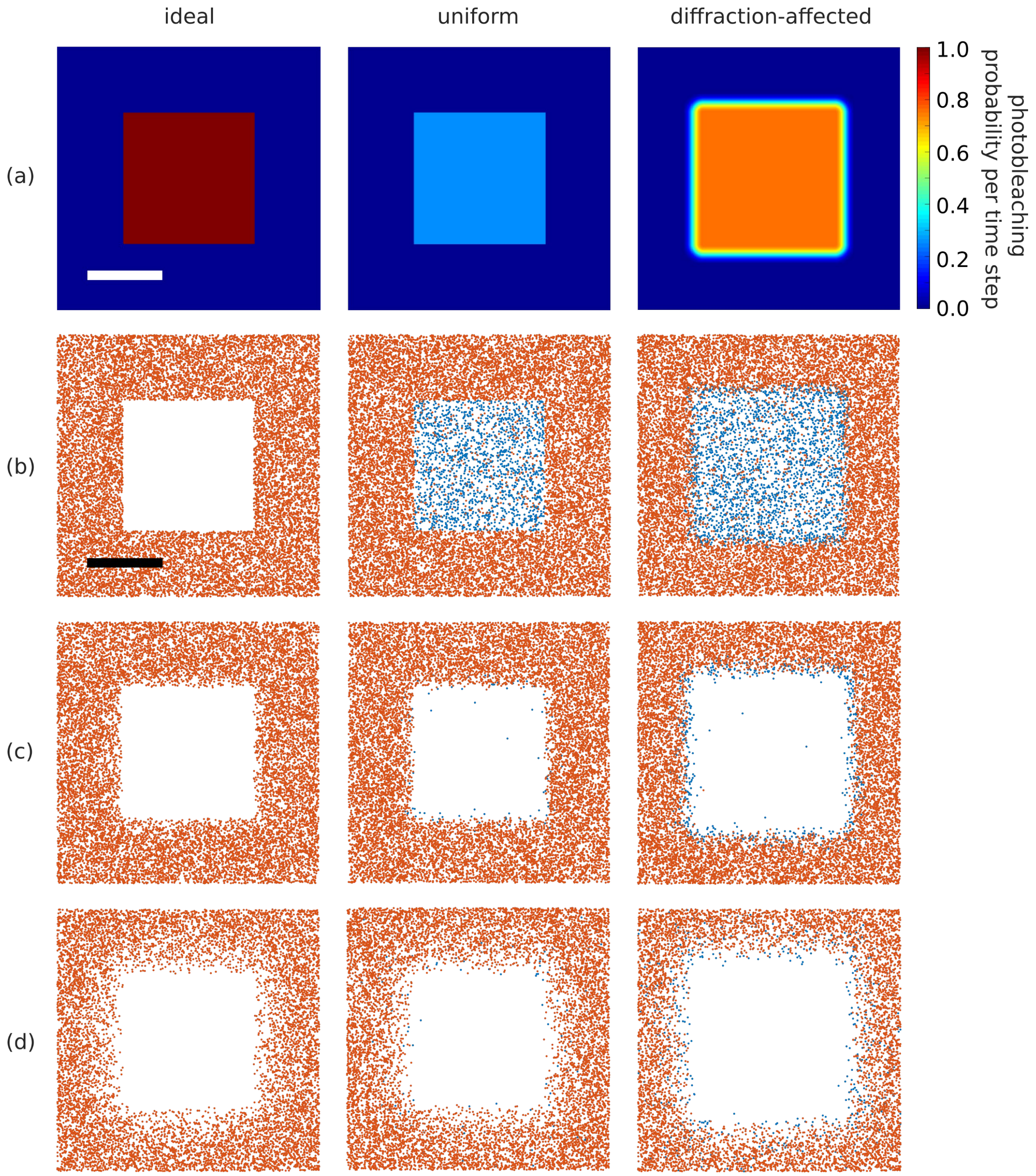

Figure S3 Shown are simulated photobleaching probability profiles for a single simulated time step (a) and exemplary simulated TOCCSL data (b-d), assuming an initial density of 100 dimers/µm² with a diffusion coefficient of $D = 0.5$ µm²/s. The central aperture-restricted region (7·7 µm^2) and the additional edge region of 1 µm (in case of the diffraction-affected laser intensity profile) are photobleached for a single time step ($t_{\mathrm{bleach}} = 0.001$ s) (a, b), for $t_{\mathrm{bleach}} = 0.25$ s (c) and for $t_{\mathrm{bleach}} = 4$ s (d), assuming ideal photobleaching (instantaneous, photobleaching with probability $p_{\mathrm{bleach}} = 1$, sharp aperture edges) (left), uniform photobleaching (time-dependent p_{bleach}, sharp aperture edges) (middle) and diffraction-affected photobleaching (time- and intensity-dependent p_{bleach}, intensity decay at the aperture edges) (right). p_{bleach} outside the illuminated area is 0. Dimers are shown in red, apparent monomers in blue. Scale bar 4 µm.

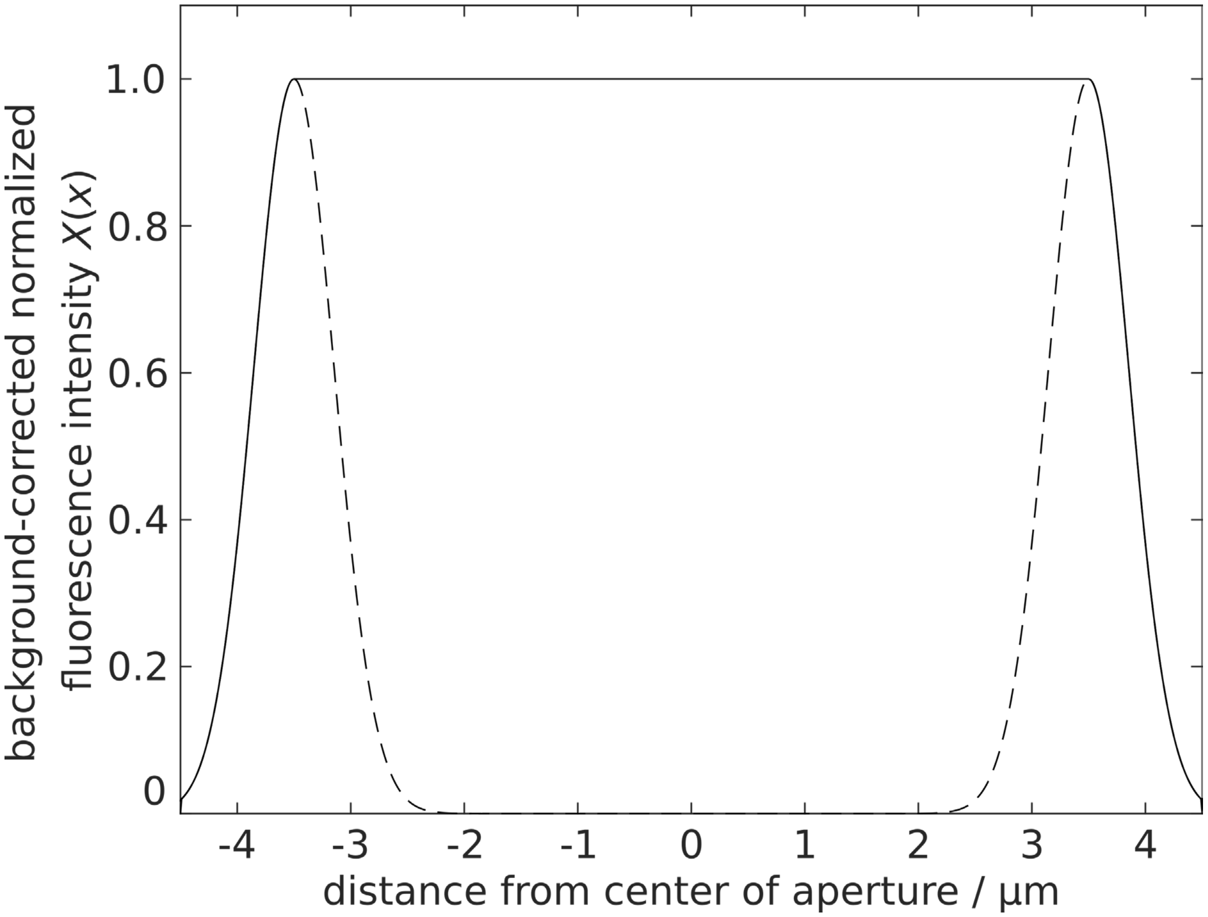

Figure S4: The intensity decay in one dimension was approximated by a Gaussian function with standard deviation $\sigma_{profile}$ = 0.5 µm and the central region by a constant plateau (black solid line). The intensity decay was assumed to reach 0 after d_{edge} = 1 µm. The two-dimensional intensity profile was obtained by multiplying the normalized intensities in *x*- and *y*-direction, $X(x)$ and $Y(y)$, yielding a normalized 2D intensity profile $I(x,y) = X(x) \cdot Y(y)$.

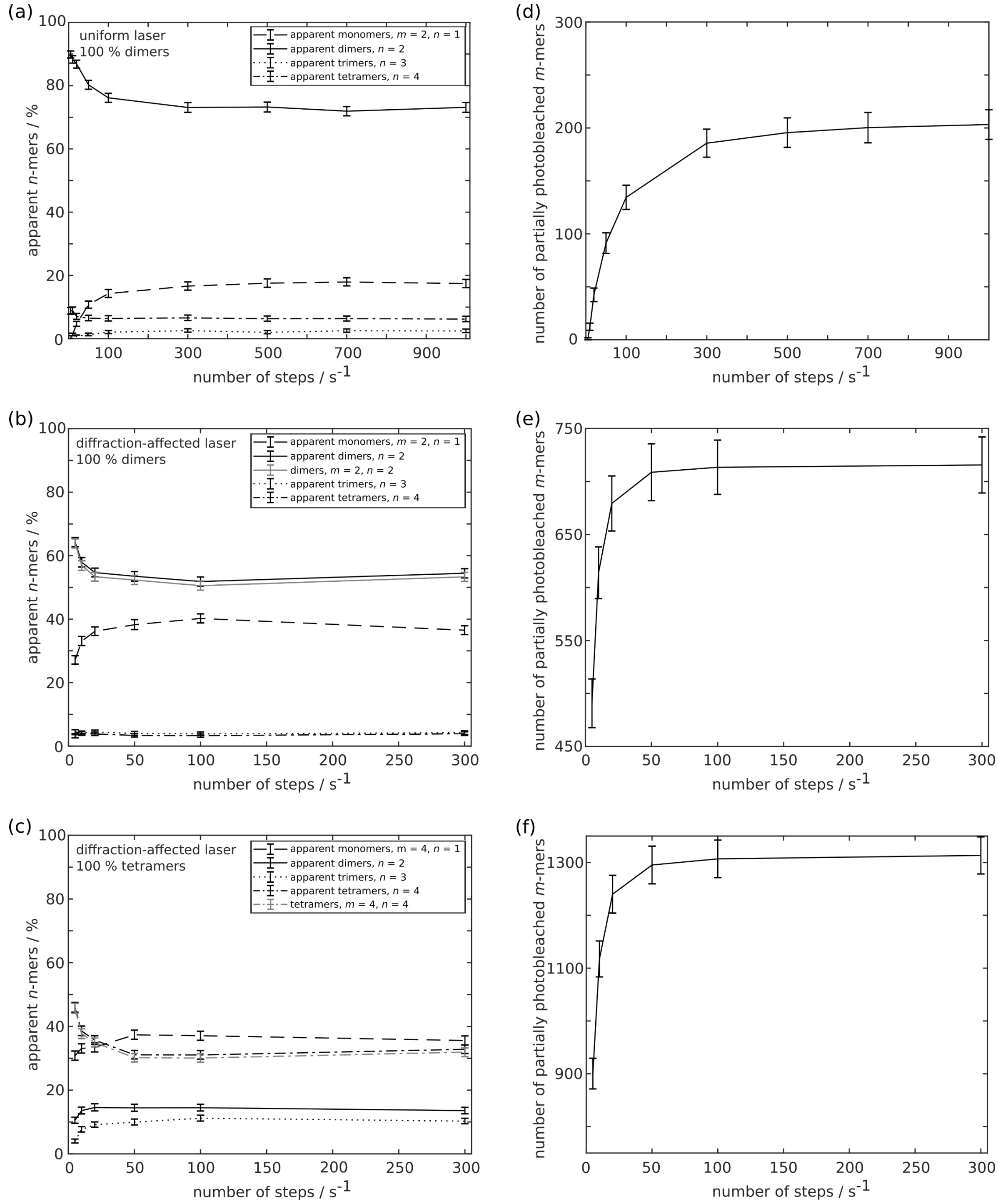

Figure S5 The accuracy of results depends on the time resolution, i.e., on the number of computational steps per simulated time-interval of 1 second. We studied here the convergence of the apparent *n*-mer fractions. A pure dimeric population (a,b,d,e) and a pure tetrameric population (c,f) with $D = 0.5$ µm²/s were exposed to photobleaching for $t_{\mathrm{bleach}} = 0.3$ s (not shown) and $t_{\mathrm{bleach}} = 4$ s assuming a uniform (a,d) or a diffraction-affected (b,c,e,f) laser intensity profile. The optimal t_{rec} and the according analysis region were determined individually for each number of steps. In the uniform case the mean fraction of apparent dimers due to random colocalizations is < 0.2 % and is omitted from the figure. For both photobleaching times, the fraction of apparent tetramers (black dashed-dotted line), the fraction of tetramers (gray dashed-dotted line), the fraction of apparent dimers (black solid line), the fraction of dimers (gray solid line) and the fraction of apparent monomers (black dashed line) do not change substantially for ≥ 300 steps per second in case of the uniform and for ≥ 50 steps per second in case of the diffraction-affected laser intensity profile. The mean number of partially photobleached *m*-mers converges for ~ 500 steps per second in case of the uniform and for ~ 100 steps per second in case of the diffraction-affected laser intensity profile. For all data a minimum of 500 steps (uniform laser intensity profile) or 100 steps (diffraction-affected laser intensity profile) per second were simulated, corresponding to a single time step of 0.002 s (uniform) or 0.01 s (diffraction-affected). Shown are the mean apparent *n*-mer fractions and the error bar (95 % bootstrapping confidence interval) (a-c) and the mean number of partially photobleached *m*-mers and the error bar (simple standard deviation) from 1000 independent simulations (d-f). The lines are a guide to the eye.

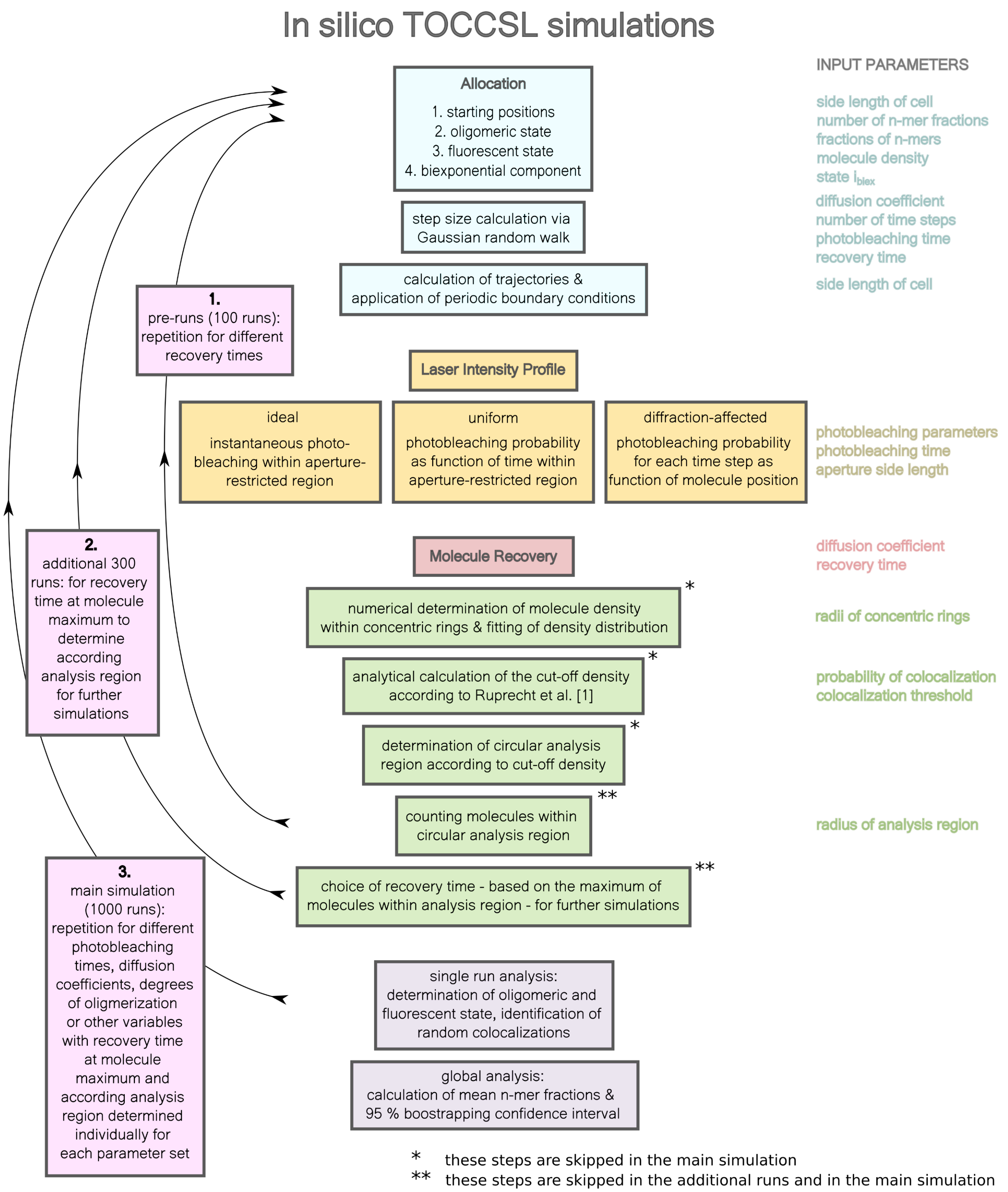

Figure S6 Coordinates x_i and y_i as well as an oligomeric and a fluorescent state are assigned to each molecule for each time step Δt. For each discrete time step Δt individual step sizes for both coordinates, Δx and Δy, are randomly drawn from a one-dimensional Gaussian probability distribution. Additionally, a state i_{biex} is assigned to each oligomeric subunit corresponding to one of two components of a biexponential photobleaching curve. To reproduce the finite size of a cell, periodic boundary conditions are applied. For an ideal, a uniform and a diffraction-affected laser intensity profile, photobleaching is simulated by pre-defining the photobleaching time and the side length of the aperture-restricted region. After photobleaching, molecules re-populate the aperture-restricted region during a pre-set recovery time. After recovery the density distribution within the photobleached area is determined. The analytically determined cut-off density (according to [1]) is compared to the density distribution. The position, at which the local density is equal to the cut-off density is determined and used for calculating the circular analysis region. Recovery is simulated for different recovery times, for each of which the analysis region and the number of molecules within is determined. The recovery time at which the highest number of molecules can be analyzed is used for further simulations, in which other parameters are varied.

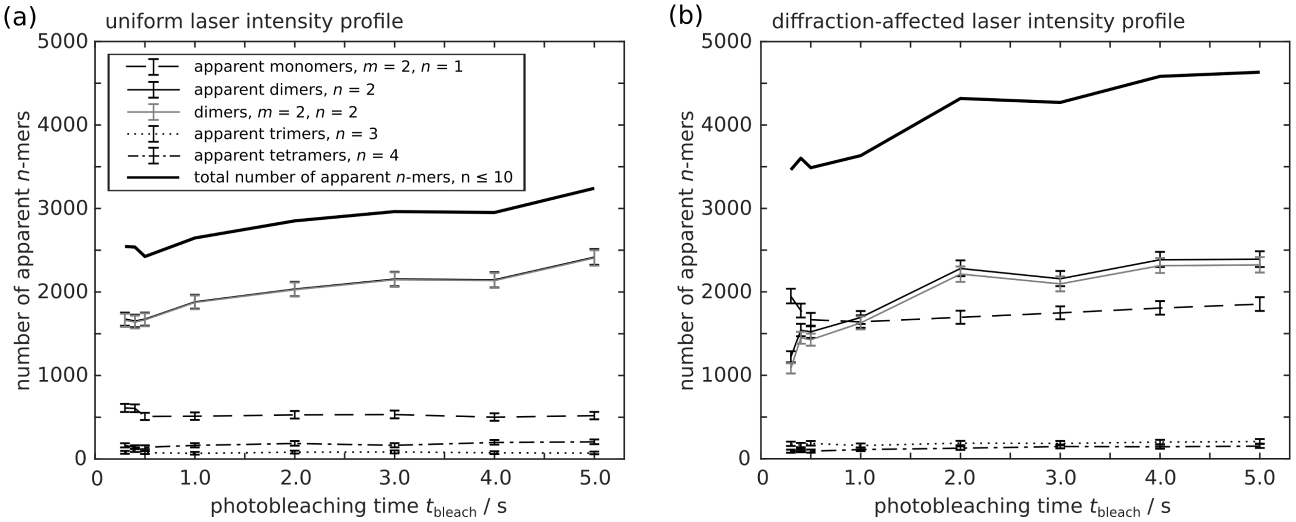

Figure S7 The influence of t_{bleach} on the number of apparent *n*-mers was studied. A purely dimeric population with D = 0.5 µm²/s was exposed to photobleaching assuming a (a) uniform or a (b) diffraction-affected laser intensity profile. According to our standard protocol, we determined for each t_{rec} an analysis region in which the molecule density is below the critical density (Eq. 7), such that the probability of two or more *n*-mers to randomly colocalize is below 20 %. For the t_{rec} at which most molecules are within the analysis region, the number of apparent *n*-mers was counted. This was done independently for different t_{bleach}. As displayed the number of recovering apparent dimers increases with increasing t_{bleach}, which translates to higher fractions of apparent dimers (Fig. 3c). Shown are the number of apparent *n*-mers (sum over 1000 simulation runs) and the error bar (95 % bootstrapping confidence interval) from 1000 independent simulations. The lines are a guide to the eye.

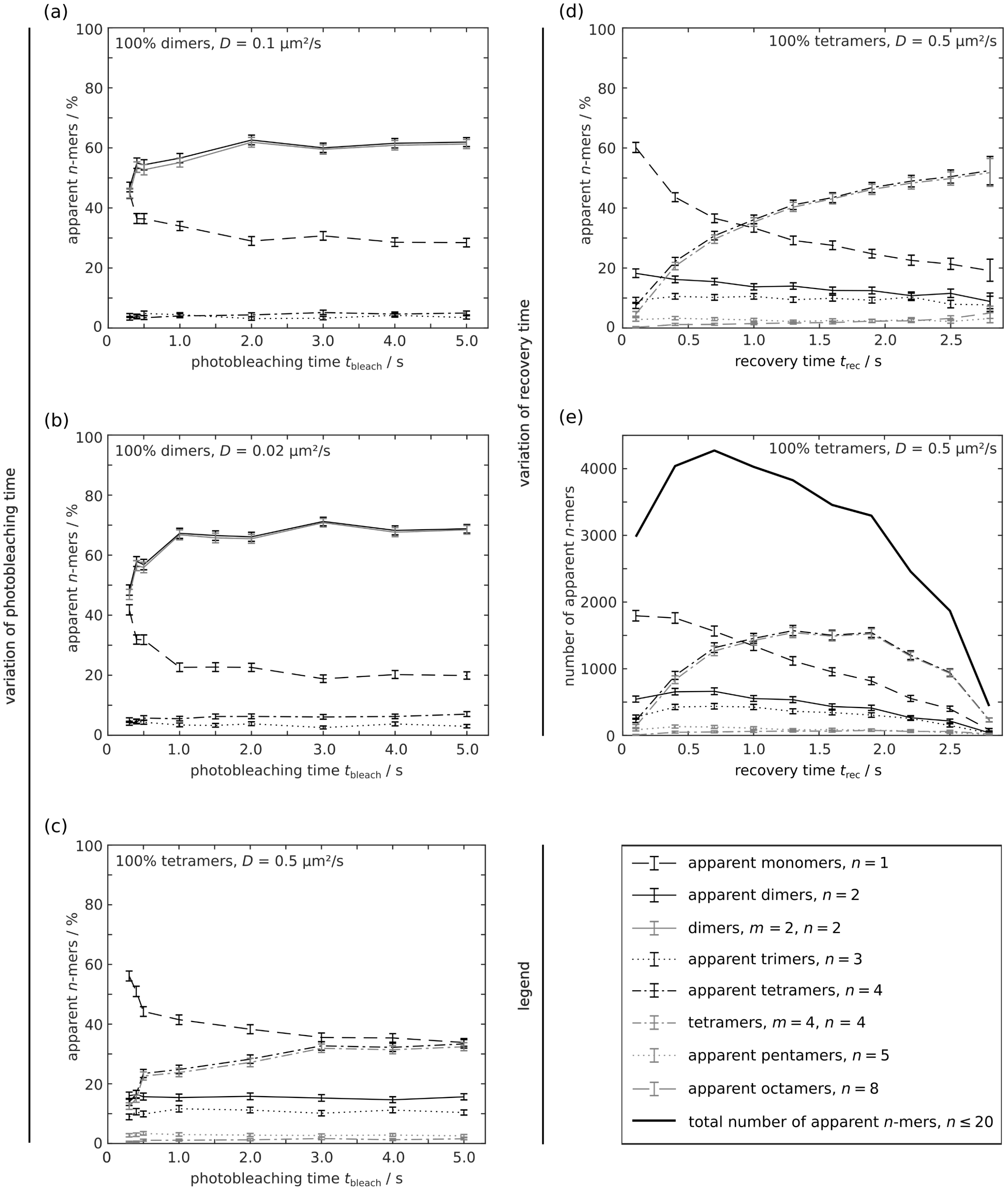

Figure S8 The influence of t_{bleach} (a-c) and t_{rec} (d-e) on the apparent *n*-mer fractions was studied. A purely dimeric population with $D = 0.1$ µm²/s (a) or $D = 0.02$ µm²/s (b) and a purely tetrameric population with $D = 0.5$ µm²/s (c-e) were exposed to photobleaching assuming a diffraction-affected laser intensity profile. The optimal t_{rec} and the according analysis region were determined individually for each t_{bleach}. With increasing t_{rec} the fraction of apparent tetramers increases (d). Shown are the mean apparent *n*-mer fractions (a-d), the number of apparent *n*-mers (sum over 1000 simulation runs) (e) and the error bar (95 % bootstrapping confidence interval) from 1000 independent simulations. The lines are a guide to the eye.

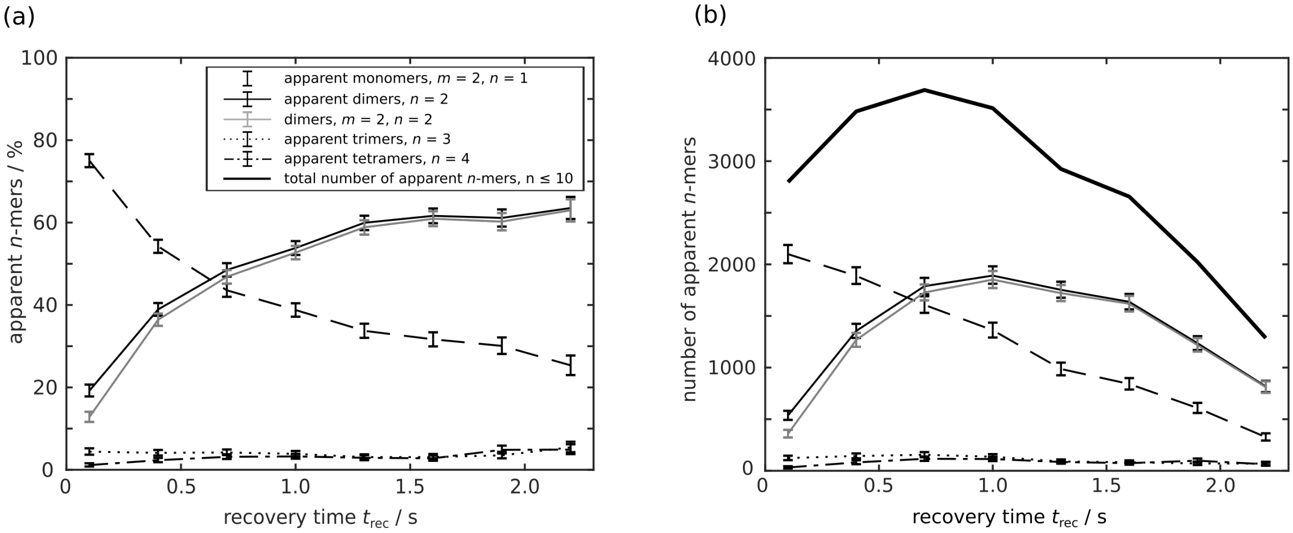

Figure S9 The influence of the shape of the aperture-restricted region was studied. A purely dimeric population with $D = 0.5$ µm²/s was exposed to photobleaching assuming a circular aperture with radius $d_{ap} = 7$ µm and a diffraction-affected laser intensity profile with an intensity decay outside the aperture-restricted region reaching 0 after $d_{edge} = 1$ µm. With increasing t_{rec} the fraction of apparent dimers increases. Shown are the mean apparent n-mer fractions and the error bar (95 % bootstrapping confidence interval) from 1000 independent simulations. The lines are a guide to the eye.

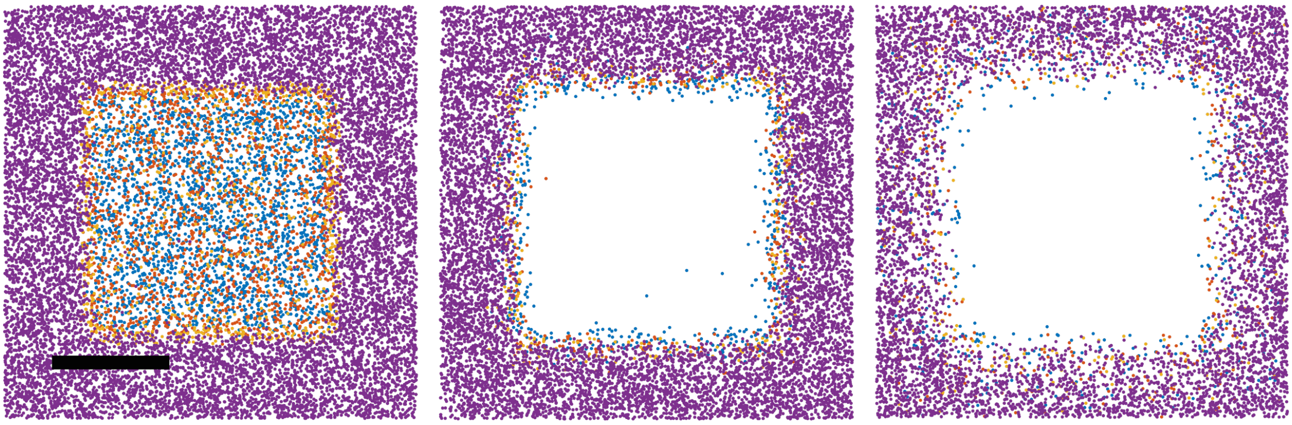

Figure S10 Shown are data from a single simulated TOCCSL experiment, assuming an initial density of 100 tetramers/µm² with a diffusion coefficient of $D = 0.5$ µm²/s and diffraction-affected photobleaching. The central aperture-restricted region (7·7 µm²) and the additional edge region of 1 µm are photobleached for a single time step ($t_{bleach} = 0.01$ s) (left), for $t_{bleach} = 0.25$ s (middle) and for $t_{bleach} = 4$ s (right). p_{bleach} outside the illuminated area is 0. Tetramers are shown in purple, apparent trimers in yellow, apparent dimers in red and apparent monomers in blue. Scale bar 4 µm.

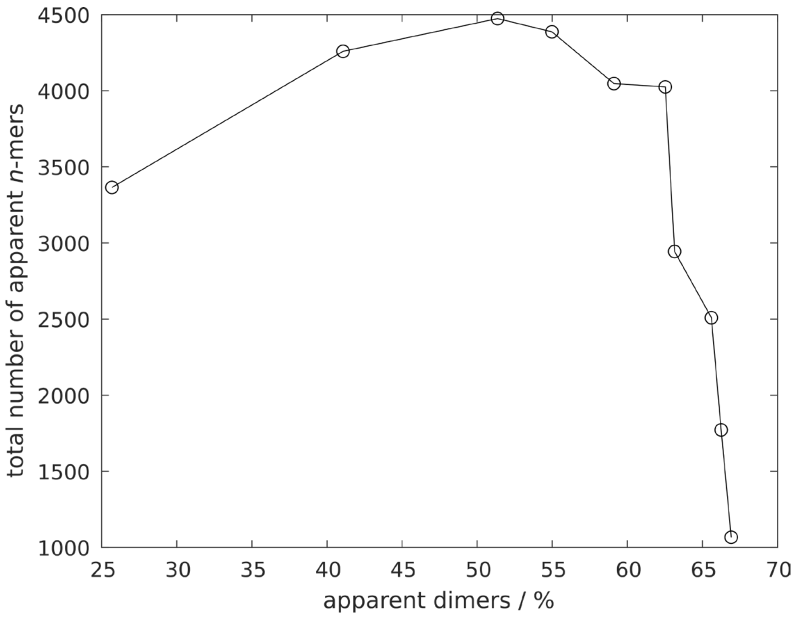

Figure S11. Shown is the total number of apparent *n*-mers within the analysis region plotted against the mean fraction of apparent dimers. A pure dimeric population with $D = 0.5$ µm²/s was exposed to photobleaching for $t_{\mathrm{bleach}} = 4$ s, assuming a diffraction-affected laser intensity profile. t_{rec} was varied between 0.1 and 2.8 s and the according analysis region was determined individually for each t_{rec}. With increasing t_{rec} the fraction of apparent dimers increases (Fig. 3d), whereas the sum of apparent *n*-mers first increases with increasing t_{rec} and then decreases for $t_{\mathrm{rec}} > 0.7$ s (Fig. 3e). To see the correlation between the apparent dimer fraction and the sum of apparent *n*-mers, data from both Fig. 3d and 3e were combined to generate the presented plot. While at the maximum number of analyzed molecules an apparent dimer fraction of ~ 51 % is determined, the maximal apparent dimer fraction of ~ 67 % is determined from a substantially lower number of analyzed molecules. The lines are a guide to the eye.

Supplemental Tables

Table S1 Fractions of apparent *n*-mers for different photobleaching times assuming a purely dimeric population of molecules and a uniform laser intensity profile.

t_{bleach} / s	apparent monomers	apparent dimers / dimers	apparent trimers	apparent tetramers
0.3	~ 24 %	~ 66 % / ~ 65 %	~ 3 %	~ 6 %
4	~ 17 %	~ 73 % / ~ 72 %	~ 3 %	~ 7 %

Table S2 Fractions of apparent *n*-mers for different photobleaching times assuming a purely dimeric population of molecules and a diffraction-affected laser intensity profile.

t_{bleach} / s	apparent monomers	apparent dimers / dimers	apparent trimers	apparent tetramers
0.3	~ 56 %	~ 35 % / ~ 31 %	~ 5 %	~ 3 %
4	~ 39 %	~ 52 % / ~ 50 %	~ 4 %	~ 3 %

Table S3 Fractions of apparent *n*-mers for different recovery times assuming a purely dimeric population of molecules and a diffraction-affected laser intensity profile.

t_{rec} / s	radius of analysis region / µm	apparent monomers	apparent dimers / dimers	apparent trimers	apparent tetramers
0.1	3.89	~ 63 %	~ 26 % / ~ 18 %	~ 8 %	~ 3 %
0.7	2.98	~ 40 %	~ 51 % / ~ 50 %	~ 4 %	~ 4 %
2.8	0.72	~ 23 %	~ 67 % / ~ 66 %	~ 4 %	~ 6 %

Table S4 Choice of recovery time and analysis region for different diffusion coefficients assuming a purely dimeric population of molecules and a diffraction-affected laser intensity profile.

diffusion coefficient / µm²/s	recovery time / s	radius of analysis region / µm
0.02	16.67	2.85
0.06	5.46	2.93
0.1	3.85	2.8
0.3	1.5	2.71
0.5	0.8	2.87

Table S5 Fraction of apparent *n*-mers for different diffusion coefficients assuming a purely dimeric population of molecules and a diffraction-affected laser intensity profile.

diffusion coefficient / µm² / s	apparent monomers	apparent dimers / dimers	apparent trimers	apparent tetramers
0.02	~ 20 %	~ 68 % / ~ 69 %	~ 4 %	~ 6 %
0.06	~ 28 %	~ 62 % / ~ 61 %	~ 4 %	~ 6 %
0.1	~ 29 %	~ 62 % / ~ 61 %	~ 4 %	~ 5 %
0.3	~34 %	~58 % / ~ 57 %	~ 4 %	~ 3 %
0.5	~39 %	~ 52 % / ~ 50 %	~ 4 %	~ 3 %

Table S6 Fraction of apparent *n*-mers for different diffusion coefficients assuming a 50 % monomeric / 50 % dimeric population of molecules and a diffraction-affected laser intensity profile.

D_2 / D_1	apparent dimers (ideal laser profile)	apparent dimers / dimers (diffraction-affected laser profile)
0.1	~ 10 %	~ 7 % / ~ 0 %
0.2	~ 8 %	~ 8 % / ~ 0 %
0.3	~10 %	~ 9 % / ~ 1 %
0.4	~12 %	~ 10 % / ~ 3 %
0.5	~ 15 %	~ 13 % / ~ 6 %
0.6	~ 23 %	~ 16 % / ~ 10 %
0.7	~ 28 %	~ 22 % / ~ 15 %
0.8	~ 35 %	~ 26 % / ~ 21 %
0.9	~ 40 %	~ 29 % / ~ 25 %
1.0	~ 47 %	~ 33 % / ~ 30 %

Table S7 Fraction of apparent *n*-mers for different diffusion coefficients assuming a purely tetrameric population of molecules and a diffraction-affected laser intensity profile.

diffusion coefficient / µm² / s	apparent monomers	apparent dimers / dimers	apparent trimers / trimers	apparent tetramers / tetramers	apparent pentamers	apparent octamers
0.02	~ 17 %	~ 7 % / ~ 7 %	~ 6 % / ~ 6 %	~ 60 % / ~ 60 %	~ 2 %	~ 4 %
50% 0.02 + 50 % 0.01	~ 22 %	~ 10 % / ~ 10 %	~ 8 % / ~ 7 %	~ 52 % / ~ 52 %	~ 2 %	~ 3 %
0.1	~ 27 %	~ 12 % / ~ 11 %	~ 9 % / ~ 8 %	~ 43 % / ~ 42 %	~ 4 %	~ 2 %
50% 0.1 + 50 % 0.5	~ 28 %	~ 13 % / ~ 13 %	~ 10 % / ~ 10 %	~ 41 % / ~ 41 %	~ 2 %	~ 2 %
0.5	~ 35 %	~ 14 % / ~ 13 %	~ 11 % / ~ 10 %	~ 33 % / ~32 %	~ 3 %	~ 2 %

References

(1) Ruprecht, V., M. Brameshuber & G. J. Schütz. 2010. Two-color single molecule tracking combined with photobleaching for the detection of rare molecular interactions in fluid biomembranes. Soft Matter 6:568-581. doi: 10.1039/B916734J
