# Supplementary material for: Monte Carlo simulations for the evaluation of oligomerization data in TOCCSL experiments
Source: Biophys J. 2023 Apr 23;122(11):2367–80. doi: 10.1016/j.bpj.2023.04.021 (PMC10257147; doi:10.1016/j.bpj.2023.04.021)
Supplement: Document S2. Article plus supporting material [file mmc2.pdf]

Biophysical Journal
Article

Biophysical Society

Monte Carlo simulations for the evaluation of oligomerization data in TOCCSL experiments

Clara Bodner,[1] Dominik Kiesenhofer,[1] Gerhard J. Schütz,[1,*] and Mario Brameshuber[1,*]
[1]Institute of Applied Physics, TU Wien, Vienna, Austria

ABSTRACT The interplay and communication between cells build the foundation of life. Many signaling processes at the cell surface and inside the cell, as well as the cellular function itself, depend on protein-protein interactions and the oligomerization of proteins. In the past, we presented an approach to single out interactions of fluorescently labeled membrane proteins by combining photobleaching and single-molecule microscopy. With this approach, termed “thinning out clusters while conserving the stoichiometry of labeling” (TOCCSL), oligomerization can be detected even at physiologically high surface densities of fluorescently labeled proteins. In TOCCSL, an aperture-restricted region of the plasma membrane is irreversibly photobleached by applying a high-intensity laser pulse. During a recovery time, in which illumination is turned off, nonphotobleached molecules from the nonilluminated area of the plasma membrane re-populate the aperture-restricted region. At the onset of this recovery process, these molecules can be detected as well-separated, diffraction-limited signals and their oligomerization state can be quantified. Here, we used extensive Monte Carlo simulations to provide a theoretical framework for quantitative interpretation of TOCCSL measurements. We determined the influence of experimental parameters and intrinsic characteristics of the investigated system on the outcome of a TOCCSL experiment. We identified the diffraction-affected laser intensity profile and the diffusion of molecules at the aperture edges during photobleaching as major sources of generating partially photobleached oligomers. They are falsely detected as lower-order oligomers and, hence, higher-order oligomers might be prevented from detection. The amount of partially photobleached oligomers that are analyzed depends on the photobleaching and the recovery time, on the mobility of molecules and—for mixed populations of oligomers—on mobility differences between different kinds of oligomers. Moreover, we quantified random colocalizations of molecules after recovery, which are falsely detected as higher-order oligomers.

SIGNIFICANCE Determination of the oligomerization state of plasma membrane proteins and lipids is key to understanding cellular signaling and function. TOCCSL (thinning out clusters while conserving stoichiometry of labeling)—an imaging modality based on single-molecule fluorescence microscopy—allows for measuring the degree of homo- or hetero-oligomerization of membrane constituents even at expression levels that are normally incompatible with single-molecule observations. In this study we determined the influence of different experimental parameters on the detected oligomerization state by utilizing Monte Carlo simulations. In silico TOCCSL experiments identified the diffraction-affected laser intensity profile and diffusion properties of the probe as major sources for influencing the determined oligomerization states. Furthermore, they allowed for optimizing experimental parameters and oligomerization data analysis.

INTRODUCTION

Assembly of biomolecules into multimolecular complexes is fundamental to cellular signaling and function. Molecular interactions lead to both the association of molecules of the same kind (homo-oligomers) and different molecular species (hetero-oligomers) and vary regarding the number of interacting molecules. Oligomers range from the smallest possible signaling unit, a molecular dimer, to larger multimolecular structures, and can host a diverse set of biomolecules.

While the importance of cell surface protein oligomerization is indisputable, there is a lack of technologies capable

Submitted August 30, 2022, and accepted for publication April 19, 2023.

*Correspondence: schuetz@iap.tuwien.ac.at or brameshuber@iap.tuwien.ac.at

Dominik Kiesenhofer's present address is Quantum Optics and Spectroscopy, Institute for Experimental Physics, University of Innsbruck, 6020 Innsbruck, Austria

Clara Bodner and Dominik Kiesenhofer contributed equally to this work.

Mario Brameshuber Twitter: @MBrameshuber

Editor: Diane Lidke.

https://doi.org/10.1016/j.bpj.2023.04.021

© 2023 Biophysical Society.

This is an open access article under the CC BY license (http://creativecommons.org/licenses/by/4.0/).

of unraveling the exact oligomeric composition of multimolecular structures on the live cell plasma membrane. Using single-molecule fluorescence microscopy, individual, fluorescently labeled cell surface proteins or protein oligomers can be directly visualized if present at low densities on the plasma membrane, e.g., via single-molecule tracking (1–3). For transmembrane receptors at physiological conditions, copy numbers of proteins range from a few molecules to 10^5 in a commonly used human cell line (4). High surface densities for more than half of these proteins cause the average distance between adjacent molecules to be smaller than the resolution limit of light microscopy, rendering direct imaging of such fluorescently labeled proteins or protein oligomers as separated diffraction-limited spots impossible. Photobleaching (5,6), induced by, e.g., high illumination laser power or illumination time, decreasing the label concentration (7), or photoactivating a subset of molecules (8) can be used to reduce the labeling density to less than one molecule per 1 μm^2, thus allowing the observation of single diffraction-limited signals. However, using these methods, the reduction of labeling density occurs stochastically and results in the absence of active labels on oligomer subunits. These subunits become invisible, thus impeding the quantification of oligomerization. Alternatively, stochastic blinking of single fluorescent molecules is used in single-molecule localization microscopy (SMLM) techniques like STORM (9,10) and PALM (11,12), thereby enabling the spatial and temporal separation of single-molecule detections. However, in these approaches different fluorophores are activated and recorded at different time points. Due to diffusion of cell surface molecules the assignment of multiple detections to one oligomeric assembly is impossible in conventional SMLM experiments. In addition, photophysics of the used fluorophores complicates quantitative counting of molecules. Although SMLM modalities such as pair-correlation PALM (13) and quantitative points accumulation in nanoscale topography (14) allow for the unbiased quantification of molecular clustering in fixed cells, they are currently not suitable for stoichiometry analysis in live cells. Dynamic super-resolution imaging of molecular interactions on spatial scales down to $\sim$30 nm was enabled by using a spectroscopic approach combining stimulated emission depletion with fluorescence correlation spectroscopy (15), allowing for probing single interactions on the milliseconds time scale. In the same line, nearfield scanning optical microscopy was combined with fluorescence correlation spectroscopy to study processes at the nanometer scale on the live cell membrane (16).

In contrast to the above-mentioned methods the single-molecule fluorescence technique Thinning out Clusters while Conserving Stoichiometry of Labeling (TOCCSL) (17) constitutes a microscopy-based strategy for studying the oligomeric composition of cell surface proteins and lipids in the plasma membrane of nonmoving, live mammalian cells and in model systems—also at physiologically high surface densities. In TOCCSL, photobleaching of fluorescent molecules is confined to a subregion of the plasma membrane by using an aperture in a sample-conjugated plane, and a high-intensity laser pulse of several kW/cm^2. During the recovery time—typically several hundred milliseconds to several seconds—molecules diffuse from the nonphotobleached area of the plasma membrane into the aperture-restricted region. In contrast to bulk fluorescence recovery after photobleaching (FRAP) experiments (18), only the beginning of the fluorescence recovery process is of interest in TOCCSL (17). Here, nonphotobleached fluorophores within the aperture-restricted region can be imaged as well-separated, diffraction-limited, single-molecule signals. TOCCSL constitutes an easy-to-implement and simple approach to determine the oligomerization state of cell surface molecules via brightness (one-color TOCCSL) (19) or co-localization (two-color TOCCSL) (20) analysis. For a more detailed explanation of TOCCSL, its applicability, and practical implementation on single-molecule microscopes see references (21,22).

In this work we characterize the influence of various experimental parameters (photobleaching time, recovery time, aperture size, size of analysis region) as well as the intrinsic characteristics of the investigated system (diffusion coefficient, oligomerization state of molecules, cell area) on the experimental outcome of a TOCCSL experiment using Monte Carlo-based in silico TOCCSL. We found that partial photobleaching of oligomers is caused by the diffraction-limited intensity decay at the aperture edges and by molecules leaving and entering the aperture-restricted region during photobleaching. Partially photobleached oligomers are detected as lower-order oligomers and, hence, higher-order oligomers might be underrepresented. In contrast, multiple molecules can randomly colocalize within the resolution limit of the optical system and be detected as a single, higher-order oligomer. While the presence of both effects can be assumed for all TOCCSL-based experiments, their magnitude was found to depend on the intrinsic parameters of the experimental systems.

METHODS

Cell culture

An GFP-GPI plasmid carrying the GPI signal sequence of the human folate receptor was constructed as described in Brameshuber et al. (19). CHO cells (ATTC CCL-61) stably expressing mGFP-GPI were continuously cultured in tissue culture flasks (GB658175; Greiner Bio-One, Kremsmünster, Austria) in MEM/HAM's F-12 medium (BE12-719F; Lonza, Basel, Switzerland) supplemented with 10% fetal calf serum (F7524; Sigma-Aldrich, St. Louis, MO), 400 μg/mL G418 (P11-012; PAA, Linz, Austria), and 100 U/mL penicillin/streptomycin (17-602E; Lonza) and maintained in a humidified incubator at 37°C and 5% CO_2 concentration.

Sample preparation

Coverglass slides (no. 1.5, BB024060SC; Menzel, Braunschweig, Germany) were glued to eight-well LabTek chambers (155411, Nunc; Thermo Fisher

Scientific, Waltham, MA) with two-component dental glue (13001001; Picodent, Wipperfürth, Germany) and washed with 70% isopropanol and sterile water. Twenty-four hours before the measurement confluent CHO cells were harvested using Accutase (00-4555-6; eBioscience, San Diego, CA), transferred to the Lab-Tek chambered coverglass slides and incubated in culture medium at 37°C and 5% CO_2 concentration. For microscopy, the culture medium was exchanged for Hanks' balanced salt solution containing calcium and magnesium (BE10-527F; Lonza) supplemented with 2% fetal calf serum. Cells were imaged at room temperature.

Microscopy

Photobleaching curves and laser intensity profiles were measured in live cells using a Zeiss Axiovert 200 inverted microscope equipped with a 100× NA = 1.46 oil immersion objective (α Plan-Apochromat; Zeiss, Oberkochen, Germany). For excitation, an optically pumped solid-state laser (Sapphire; Coherent, Santa Clara, CA) with a wavelength of 488 nm and a laser power of $\sim$5 kW/cm^2 in the sample plane was used. The illumination area was restricted by a rectangular aperture placed in a sample-conjugated plane. For exact timings, an acousto-optic modulator (Isomet, Springfield, VA) was used. Timing protocols were created and controlled by an in-house-written program package implemented in LABVIEW (National Instruments, Austin, TX). An appropriate dichroic mirror (ZT488/640rpc 2 mm; Chroma, Bellows Falls, VT) and an emission filter (FF01-538/685-25; Semrock, Rochester, NY) were used.

Photobleaching curves

For reducing the cytosolic background, cells were prebleached in non-TIRF configuration. For each cell a sequence of 200 images was recorded in objective-based total internal reflection (TIR) configuration with an EMCCD camera (Andor iXon Ultra DU-897; Oxford Instruments, Abingdon, UK). We used stroboscopic illumination with an illumination time of 1 ms and a time lag of 7 ms between consecutive images. The overall photobleaching time is hence given by the cumulative illumination time of $200 \cdot 1$ ms = 200 ms and was sufficient to deplete the region of interest (ROI) of the cell surface from all active fluorophores. All sequences were analyzed in ImageJ.

Simulation of diffusing molecules

The simulation software is implemented in MATLAB R2019a (9.6.0.1072779, 64-bit; The MathWorks, Natick, MA) on a personal computer running Windows 10 (Microsoft, Redmond, WA). All scripts are available under https://github.com/schuetzgroup/In_silico_TOCCSL.git. Diffusing fluorescently labeled molecules are simulated for an area of $21 \cdot 21$ μm^2 with a density of 100 molecules/μm^2. In the following, this simulated quadratic area is termed "cell area." Importantly, all presented results are independent of the cell area for cell side lengths larger or equal to 14 μm (Fig. S1, *a* and *b*). For smaller cell areas, depletion of fluorescent molecules influences the outcome of a TOCCSL experiment. The coordinates x and y of each molecule's starting position are randomly drawn from a uniform distribution on the interval [0, 21]. For each discrete time step Δt, individual step sizes for both coordinates, Δx_i and Δy_i, are randomly drawn from a one-dimensional Gaussian probability distribution with variance $\sigma^2 = 2\,D\,\Delta t$ and mean $\mu = 0$. Gaussian distributed random numbers are generated with the built-in normrnd function (MATLAB). New coordinates x_{i+1} and y_{i+1} are assigned to each molecule for each time step Δt by adding the randomly drawn step sizes Δx_i and Δy_i to the former molecule coordinates x_i and y_i:

$$x_{i+1} = x_i + \Delta x_{i+1} \qquad \text{(Equation 1a)}$$

$$y_{i+1} = y_i + \Delta y_{i+1}\,. \qquad \text{(Equation 1b)}$$

This is done for the total number of steps and all molecules in the simulation. Each molecule's coordinates at a time point t are given by the cumulative sum of its starting position and all spatial steps the diffusing molecule has taken during t. To preserve the initial density of molecules, periodic boundary conditions are applied. If a molecule passes through one side of the interval [0, 21] in x or y direction, it reenters it on the opposite side. In the case of mixed populations with oligomers of different order and with different diffusion coefficients D, the probability distribution for each population differs according to the variance of the one-dimensional Gaussian function. Thus, the calculation of the molecules' trajectories is done separately for each oligomeric composition.

Oligomeric, fluorescent, and photobleaching state

Each molecule exhibits a predefined oligomeric state m (number of molecule subunits) and a separate fluorescent state n (number of molecule subunits carrying an active fluorophore). Oligomeric and fluorescent state are assigned to every molecule and for every time step Δt they are stored along with their trajectory coordinates. For simplicity, a labeling degree and an ideal labeling efficiency of 1 are assumed. The oligomeric state remains unaltered during the simulation, while the fluorescent state, which initially equals the oligomeric state, can change during the simulated photobleaching process. Transient oligomerization (dissociation and recombination) of proteins is not considered. Molecules with oligomeric state m and fluorescent state n are henceforth denoted as m-mers and apparent n-mers. m-mers with fluorescent state $n = 0$ after photobleaching are denoted as fully photobleached m-mers, m-mers with fluorescent state $0 < n < m$ after photobleaching as partially photobleached m-mers, and m-mers with fluorescent state $n = m$ after photobleaching as nonphotobleached m-mers. In addition, a state i_{biex} is assigned to each oligomeric subunit corresponding to one of two components of a biexponential photobleaching curve, as described in the next section. This assignment remains unaltered in the course of the photobleaching process.

Simulation of photobleaching

Photobleaching of fluorophores due to the exposure to a high-energy light source (e.g., a laser) depends on the illumination intensity at the sample plane as well as the time t_{ill} that molecules reside within the aperture restricted, illuminated area during the photobleaching process. Importantly, the choice of the aperture side length d_{ap} influences the outcome of a TOCCSL experiment (Fig. S1, *c–e*). For all simulations shown, a quadratic aperture-restricted region of $d_{\mathrm{ap}} \cdot d_{\mathrm{ap}} = 7 \cdot 7$ μm^2 is employed. For molecules that diffuse in and out of the illuminated area during the photobleaching time t_{bleach}, t_{ill} is shorter than t_{bleach}. To characterize the photobleaching probability p_{bleach} of fluorophores in TOCCSL experiments, photobleaching curves of CHO mGFP-GPI cells were recorded as described above. For each image sequence, a ROI within the illuminated area was selected manually and applied to all 200 images. The integrated ROI brightness over time was background corrected, normalized, and fitted via linear least squares by a two-component exponential function

$$b(t_{ill}) = A' e^{-\alpha t_{\mathrm{ill}}} + B' e^{-\beta t_{\mathrm{ill}}} \qquad \text{(Equation 2)}$$

with α and β denoting the photobleaching parameters and A' and B' the weights of the two fit components (Fig. S2). For simulating the photobleaching process, the photobleaching curve fits were determined for eight independent cells. The mean values of the fit parameters were used for further calculations and yielded $\alpha = 235$ 1/s and $\beta = 30$ 1/s. Normalized weights $A = A'/(A' + B') = 0.782$ and $B = B'/(A' + B') = 0.218$ were introduced such that $A + B = 1$.

To reproduce the biexponential photobleaching behavior in the simulations, one of the two biexponential components ($i_{\mathrm{biex}} = 1$ for α or $i_{\mathrm{biex}} = 2$ for β) is assigned to each oligomeric subunit according to their weights A and B in the biexponential photobleaching curve. Each of the two species

follows a photobleaching process governed by one of the coefficients α or β. The photobleaching probability for a normalized laser intensity of 1 is calculated as

$$p_{\text{bleach}}(\Delta t) = 1 - \left(e^{-\alpha \Delta t}\right) \quad \text{for} \quad i_{\text{biex}} = 1 \qquad \text{(Equation 3a)}$$

$$p_{\text{bleach}}(\Delta t) = 1 - \left(e^{-\beta \Delta t}\right) \quad \text{for} \quad i_{\text{biex}} = 2. \qquad \text{(Equation 3b)}$$

Outside the illuminated area, p_{bleach} was assumed to be 0. Three different laser intensity profiles were studied: 1) an ideal laser intensity profile (Fig. S3, *left*) with photobleaching probability $p_{\text{bleach}} = 1$ for each time step Δt within the aperture-restricted region $d_{\text{ap}} \cdot d_{\text{ap}}$. 2) A uniform laser intensity profile (Fig. S3, *middle*) with p_{bleach} following Eq. 3 for each time step Δt within the aperture-restricted region $d_{\text{ap}} \cdot d_{\text{ap}}$. 3) A realistic, diffraction-affected laser intensity profile (Fig. S3, *right*) exhibiting a uniform laser intensity profile within the aperture-restricted region $d_{\text{ap}} \cdot d_{\text{ap}}$ and an intensity decay outside the aperture edges reaching zero after $d_{\text{edge}} = 1$ μm. The diffraction-induced intensity decay in one dimension was approximated by a Gaussian function with standard deviation $\sigma_{\text{profile}} = 0.5$ μm and the central region by a plateau (Fig. S4). The relative laser intensity $X(x)$ with respect to the maximum value at the plateau is specified for the absolute value of the distance x to the center of the illuminated region by

In the case of the diffraction-affected laser intensity profile, the updated fluorescent state is assigned to the residual part of the molecule's trajectory after each time step Δt during t_{bleach}. Hence, the new fluorescent state constitutes the basis for the next photobleaching step.

For both the uniform and the diffraction-affected laser intensity profile, stochastic photobleaching is simulated using a Monte Carlo method for determining whether a fluorescent molecule is dark or remains fluorescent after residing inside the aperture-restricted region for a duration of t_{ill}. This is done independently for all m molecule subunits of each oligomeric complex of order m. The individual photobleaching probability on the interval [0, 1] is calculated as described above depending on the choice of the laser intensity profile. The resulting value is then compared with a uniformly distributed random number generated with the built-in *rand* function (MATLAB) on the interval [0, 1]. If the photobleaching probability is larger than or equal to the generated random number the molecule is irreversibly photobleached, and, thus, the molecule's fluorescent state n is reduced by one. In summary, the photobleaching probability for a single oligomeric subunit follows a monoexponential decay, for both the uniform and the diffraction-affected laser intensity profiles. The average photobleaching probability considering all oligomers in the illuminated area follows a biexponential decay as observed experimentally. A minimal time step of $\Delta t = 0.002$ s (uniform laser intensity profile) or $\Delta t = 0.01$ s (diffraction-affected laser intensity profile) was sufficient to determine p_{bleach} without altering the outcome of simulated TOCCSL experiments (Fig. S5).

$$\begin{aligned}
X(|x|) &= 1 && \text{for } |(x)| \nleqq \frac{d_{\text{ap}}}{2} \\
X(|x|) &= \exp\left[\frac{-\left(|x| - \left(\frac{d_{\text{ap}}}{2}\right)\right)^2}{\sigma_{\text{profile}}^2}\right] && \text{for } \frac{d_{\text{ap}}}{2} + d_{\text{edge}} \nleqq |(x)| > \frac{d_{\text{ap}}}{2} \\
X(|x|) &= 0 && \text{for } |(x)| > \frac{d_{\text{ap}}}{2} + d_{\text{edge}}\,.
\end{aligned} \qquad \text{(Equation 4)}$$

The two-dimensional intensity profile was obtained by multiplying the relative intensities in x and y directions, $X(x)$ and $Y(y)$, with each other, yielding a normalized two-dimensional intensity profile $I(x,y) = X(x) \cdot Y(y)$. In the case of the diffraction-affected laser intensity profile the photobleaching parameters depend on the laser intensity profile $I(x,y)$ and yield $\alpha_{\text{I}} = \alpha \cdot I(x,y)$ and $\beta_{\text{I}} = \beta \cdot I(x,y)$. For each time step Δt during t_{bleach}, the coordinates of all molecules are queried and p_{bleach} is determined separately for each molecule as a function of the laser intensity profile $I(x,y)$ at the exact molecule position within the illuminated area $[d_{\text{ap}} + 2 \cdot d_{\text{edge}}] \cdot [d_{\text{ap}} + 2 \cdot d_{\text{edge}}]$ ($9 \cdot 9$ μm^2). Considering $I(x,y)$, i_{biex} α and β, the probability of a fluorescent molecule with coordinates x and y to be irreversibly photobleached after a single time step Δt can be calculated as

$$p_{\text{bleach}}(x, y, \Delta t) = 1 - \left(e^{-\alpha I(x,y)\Delta t}\right) \quad \text{for} \quad i_{\text{biex}} = 1 \qquad \text{(Equation 5a)}$$

$$p_{\text{bleach}}(x, y, \Delta t) = 1 - \left(e^{-\beta I(x,y)\Delta t}\right) \quad \text{for} \quad i_{\text{biex}} = 2. \qquad \text{(Equation 5b)}$$

Consideration of random colocalizations

After photobleaching, fluorescently labeled molecules from outside the aperture-restricted region diffuse back into the dark, photobleached area during a preset recovery time, where they are analyzed regarding their oligomeric state m and fluorescent state n. A priori, all recovered molecules are considered for stoichiometry analysis. In a real single-molecule microscopy experiment two adjacent molecules imaged by their point spread functions (pdf) cannot be distinguished from each other if their mutual distance is smaller than the resolution limit of light (Rayleigh criterion). In our simulations molecules are considered point-like objects with well-defined coordinates. However, the Rayleigh criterion is applied to account for diffraction-limited resolution in real experiments. Two or more molecules with fluorescent state n_1, n_2, n_3, … within a threshold distance R from each other are counted as one "apparent n-mer" ($n = \Sigma_i n_i$). This also applies if one or more molecules lie outside the analysis region but within the distance R of a molecule inside the analysis region. Apparent n-mers due to random colocalizations are only considered for $n \leq 5 \cdot m_{\text{max}}$, with m_{max} being the largest initially assigned oligomeric fraction. The right choice of R generally depends on the intermolecular subunit distances of the observed molecule, the emission wavelength of the fluorophore, the

resolution of the microscopy setup, and the quality of image analysis. For all simulations presented, R was set to 300 nm. The detection of apparent n-mers due to random colocalizations results in an overestimation of the fraction of higher-order oligomers and an underestimation of the fraction of lower-order oligomers. As the accidental coincidence of molecules increases with higher molecule density, the fraction of apparent n-mer detections due to random colocalizations is directly related to the molecule density ρ. If all molecules are randomly distributed over the cell area, the probability for one of them to be located within a radius R of another molecule is given by

$$p_{\mathrm{coloc}} = 1 - e^{\left(-R^2\pi\rho\right)} \ (20). \qquad \text{(Equation 6)}$$

Since the recovery of molecules after photobleaching proceeds from the aperture edges, the density ρ and, hence, p_{coloc} increases with increasing distance from the center of the photobleached region. To keep p_{coloc} low, the area confining the number of analyzed molecules has to be limited to a central region within the illuminated area that exhibits a maximum molecule density equal to the cutoff density

$$\rho_{\mathrm{crit}} = -\ln\frac{\left(1 - p_{\mathrm{coloc}}\right)}{R^2\pi}. \qquad \text{(Equation 7)}$$

For all simulations presented, a maximum p_{coloc} of 20% was accepted, yielding a mean p_{coloc} of ~10–20%. ρ_{crit} was calculated to be 0.79 molecules/μm^2.

Data analysis

Each mean fraction of apparent n-mers, f_n was calculated from 1000 independent simulation runs by

$$f_n[\%] = \frac{v_n}{\sum_{n=1}^{5m_{max}} v_n} \cdot 100 \qquad \text{(Equation 8)}$$

with v_n being the number of apparent n-mers inside the analysis region (sum over 1000 simulation runs), including events that are due to randomly colocalized molecules. Each mean fraction of m-mers f_m was calculated from 1000 independent simulation runs by

$$f_m[\%] = \frac{v_m}{\sum_{n=1}^{5m_{max}} v_n} \cdot 100 \qquad \text{(Equation 9)}$$

with v_m being the number of m-mers inside the analysis region (sum over 1000 simulation runs), excluding events that are due to randomly colocalized molecules.

The 95% bootstrapping confidence interval was determined by random sampling with replacement. One sample was created by randomly drawing 1000 times from the 1000 simulation runs. From 10,000 samples the 95% confidence interval of each apparent n-mer fraction was determined for each parameter set.

RESULTS

Simulation of TOCCSL experiments

An overview of the methodical workflow is shown in Fig. S6. In silico TOCCSL experiments are initiated by randomly placing single molecules within an area of 21 · 21 μm^2 at a density of 100 molecules/μm^2 (Fig. 1, *left*). Two-dimensional Brownian motion of molecules is realized via Monte Carlo simulations of random walkers

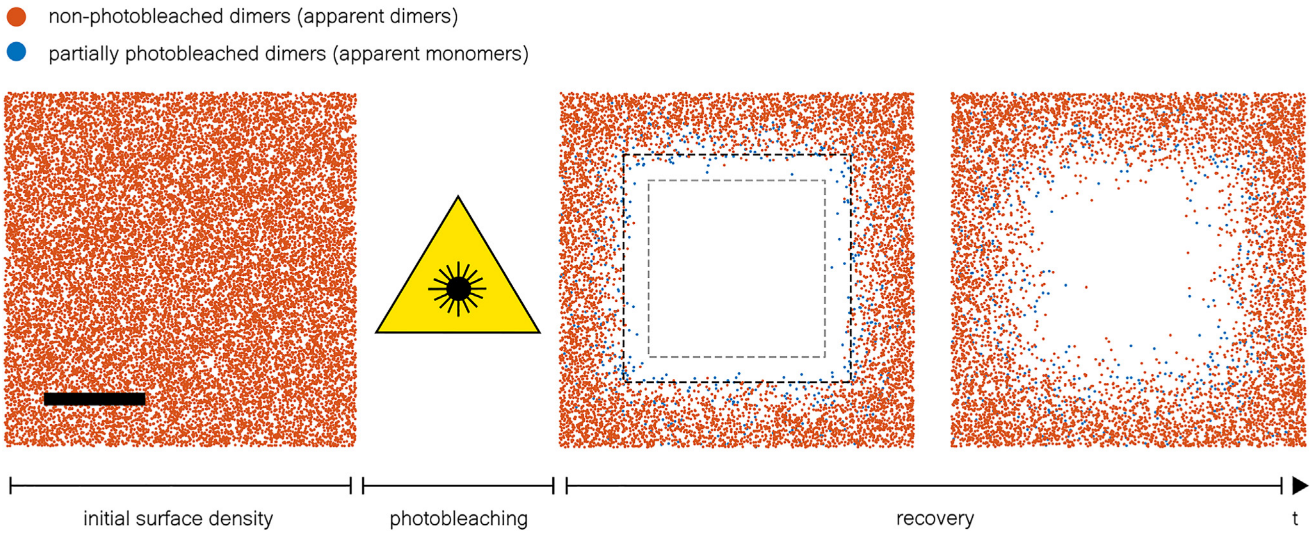

FIGURE 1 In silico TOCCSL experiment. A total cell area of 21 · 21 μm^2 was simulated. The left image shows the central region of the simulated cell with initial density of 100 dimers/μm^2. The photobleaching process is simulated assuming a diffraction-affected laser intensity profile that exhibits a uniform laser intensity profile within the aperture-restricted region (*gray dashed lines*) and a diffraction-induced intensity decay at the aperture edges that reaches 0 after 1 μm (*black dashed lines*). After photobleaching, the center of the aperture-restricted region exhibits no active fluorophores (*middle*). Due to diffraction effects at the aperture edges and dimers leaving and entering the illuminated area during t_{bleach} some of them undergo partial photobleaching. Partially photobleached and nonphotobleached dimers from outside the illuminated area diffuse into the photobleached region during a recovery time t_{rec} (*right*). For the TOCCSL simulation shown a diffusion coefficient of $D = 0.5$ μm^2/s, a photobleaching time of $t_{\mathrm{bleach}} = 4$ s, and a $t_{\mathrm{rec}} = 0$ s (*middle*) or $t_{\mathrm{rec}} = 0.8$ s (*right*) were used. The figures display the central region of the simulated area. Scale bar, 4 μm.

with fixed oligomeric state m (termed m-mers); n denotes the number of active fluorophores associated with the oligomer as a measure of the fluorescent state (termed apparent n-mers). In our study we simulated purely dimeric ($m = 2$) or purely tetrameric ($m = 4$) samples, or samples containing a 1:1 mixture of monomeric ($m = 1$) and dimeric ($m = 2$) molecules. Oligomeric and fluorescent states are assumed to be equal prior to photobleaching, i.e., $m = n$. A central rectangular area (indicating the aperture-restricted region) is photobleached for a duration of t_{bleach} (see simulation of photobleaching), thus altering the fluorescent state of molecules that are exposed to photobleaching. Molecules that leave and enter the aperture-restricted region during t_{bleach} are illuminated for a duration of $t_{ill} < t_{bleach}$. Fig. 1 (*middle*) shows an example of dimers with diffusion coefficient $D = 0.5\ \mu m^2/s$ and their respective position and fluorescent state directly after photobleaching for $t_{bleach} = 4$ s.

To distinguish the effects of different error sources on the obtained apparent oligomerization states, we simulated the photobleaching process for three different scenarios: 1) an ideal laser intensity profile (Fig. S3, *left*) with photobleaching probability $p_{bleach} = 1$ within the aperture-restricted region, 2) a uniform laser intensity profile (Fig. S3, *middle*) with p_{bleach} following Eq. 3, and 3) a diffraction-affected laser intensity profile (Fig. S3, *right*) exhibiting a uniform laser intensity profile within the aperture-restricted region and a diffraction-induced intensity decay at the aperture edges. For each time step during t_{bleach}, p_{bleach} depends on the laser intensity $I(x,y)$ at the molecules' position (Eq. 5, also see simulation of photobleaching).

After the photobleaching pulse, molecules from outside the aperture-restricted region diffuse into the photobleached area during a recovery time t_{rec}. Fig. 1 shows an example of the recovery process directly after photobleaching ($t_{rec} = 0$ s, *middle*) and after $t_{rec} = 0.8$ s (*right*). Within a subregion of the photobleached area, recovered molecules are analyzed with respect to their fluorescent state.

Choice of recovery time and analysis region

The choice of t_{rec} directly influences the density distribution of recovered molecules within the aperture-restricted region. The longer t_{rec}, the more molecules can diffuse into the photobleached area, which leads to an increase of the molecule density ρ. If—after t_{rec}—two or more molecules with fluorescent state n_1, n_2, n_3, … randomly colocalize within 300 nm, they are counted as one apparent n-mer ($n = \Sigma_i n_i$). According to Eq. 6, the probability for random colocalizations, p_{coloc}, increases with increasing ρ. Since molecules recover from outside the aperture edges, ρ and consequently also p_{coloc} increase in radial direction with increasing distance from the center within the photobleached area. To keep p_{coloc} below a threshold (in our study we chose 20%), only a region for which ρ is equal to the cutoff density ρ_{crit} (Eq. 7) is considered, yielding a mean p_{coloc} of 10–20% for all simulations shown.

In our simulations we selected the recovery time based on the maximum of analyzed molecules. For this purpose, the recovery process of dimers after photobleaching was simulated for different t_{rec} (Fig. 2). The density distribution after recovery (Fig. 2 *c*) was determined by sectioning the aperture-restricted region into concentric rings with outer radii from 10 nm to 1.5 times the aperture side length d_{ap} in 10 nm steps around the center of the photobleached area (Fig. 2, *a* and *b*) and dividing the number of molecules within each concentric ring (mean from 100 independent simulations) by the area of the respective ring. A circular analysis region was chosen, as the density of recovered molecules is higher in the corners of the aperture-restricted rectangular region and the density distribution determined from a rectangular analysis region would not be representative for the actual local densities. Between 0 and $(d_{ap} + d_{edge})/2$, the resulting local density values were fitted by

$$\rho(r) = \rho_0 \cdot \left(1 - \frac{1}{2}\cdot \mathrm{erf}\left(\frac{r + \frac{d'}{2}}{\sqrt{4D't'}}\right) + \frac{1}{2}\cdot \mathrm{erf}\left(\frac{r - \frac{d'}{2}}{\sqrt{4D't'}}\right)\right) \quad \text{(Equation 10)}$$

with erf denoting the error function, r the distance from the center of the aperture-restricted region, and ρ_0, d', and $\tau = \sqrt{4D't'}$ the fitting parameters (adapted from (20)). We determined the largest radius $r_{analysis}$ for which $\rho(r_{analysis}) = \rho_{crit}$ and used this value to calculate the area of the circular analysis region. Fig. 2 *d* shows the number of molecules inside the analysis region for different t_{rec}. A cubic polynomial fit was used to determine the position of the maximum, which corresponds to the optimal t_{rec}. For the optimal t_{rec}, the according analysis region was determined from 300 additional simulations.

For all presented simulations, in which other parameters (t_{bleach}, diffusion coefficient D, oligomeric state m, aperture side length d_{ap}, cell area) were varied, the optimal t_{rec} and the according analysis region were determined individually and applied to 1000 independent simulations. Recovered molecules within the analysis region were analyzed with respect to their fluorescent state and potential random colocalizations, and the fractions of apparent n-mers were calculated (mean of 1000 simulations).

Influence of photobleaching time on the apparent dimer fraction

The choice of t_{bleach} directly influences the photobleaching probability p_{bleach} of molecules that are residing within the aperture-restricted region, as described in simulation of photobleaching (in the case of a uniform or a diffraction-affected laser intensity profile). We investigated the influence

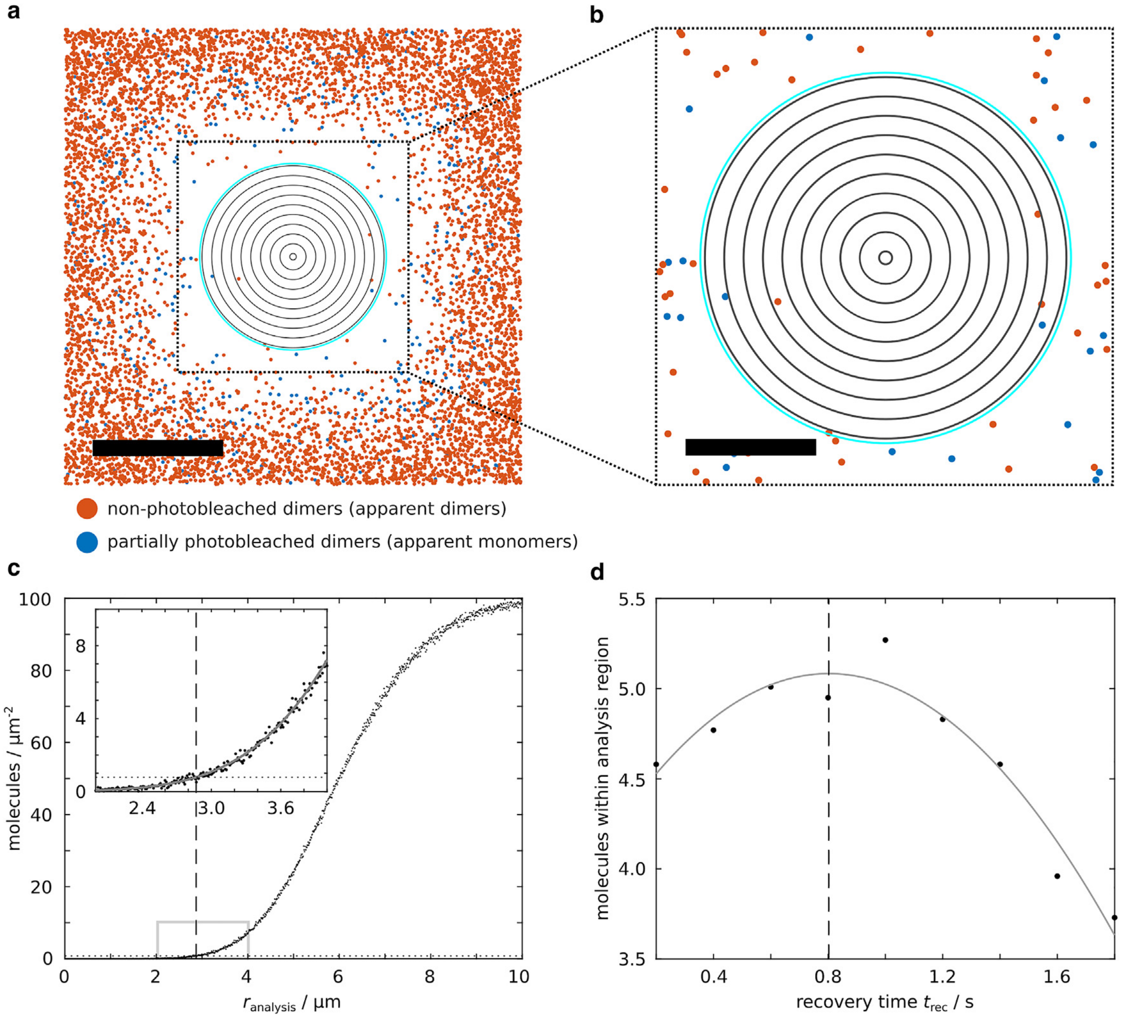

FIGURE 2 Choice of recovery time and analysis region. (*a*) An aperture-restricted region (*black dashed box*) and a diffraction-affected edge region (1 µm on each side of the aperture) are exposed to photobleaching for $t_{bleach} = 4$ s, assuming a diffraction-affected laser intensity profile. Shown are dimers ($m = 2$) with $D = 0.5$ µm^2/s after recovery ($t_{rec} = 0.8$ s). Starting from the center, the photobleached region is sectioned into concentric rings with outer radii from 10 nm to $1.5 \cdot d_{ap}$ in 10 nm steps (*gray rings*, here only every 30th ring is shown). The cyan ring denotes the chosen radius of the analysis region, $r_{analysis}$, based on the cutoff density ρ_{crit} (Eq. 7). Scale bar, 4 µm. (*b*) Zoom into the black boxed region. Scale bar, 2 µm. (*c*) The mean number of molecules (with fluorescent state $0 < n \leq m$) per ring area is determined from 100 independent simulations, yielding the local density within each ring (*black dots*). The resulting density distribution along the radial direction is fitted (*gray solid curve*) and compared with ρ_{crit} (*black dotted line*), which is calculated for the colocalization threshold distance $R = 300$ nm between two molecules and the maximum probability of random colocalizations, $p_{coloc} = 20\%$. From the density distribution fit, the outer radius of the concentric ring $r_{analysis}$ that exhibits the highest molecule density $\rho\,(r_{analysis}) = \rho_{crit}$ is determined (*black dashed line*), which is used to calculate the area of the analysis region. The inset shows the zoom into the gray boxed region. (*d*) For each t_{rec} the analysis region is calculated as outlined in (*c*) and the mean numbers of molecules within are determined. The optimal t_{rec}, at which most molecules can be analyzed (*black dashed line*), is determined from a cubic polynomial fit (*gray solid line*). A cubic polynomial fit is chosen, as the aim of the fit is not to represent the exact number of molecules for any t_{rec}, but solely to find the maximum of molecules. The optimal t_{rec} and the according analysis region are used for further simulations.

of t_{bleach} on the outcome of a TOCCSL experiment, assuming a purely dimeric population of molecules ($m = 2$) with $D = 0.5$ µm^2/s. For each of the three different laser intensity profiles (ideal, uniform, diffraction-affected), in silico TOCCSL experiments were performed. A central region was photobleached for t_{bleach} between 0.3 and 5 s. Fig. 3, *a–c*, display each fraction of apparent *n*-mers inside the analysis region after recovery, for the three different laser profiles.

In the case of an ideal laser intensity profile, p_{bleach} is equal to 1 for each time step and, hence, independent of t_{bleach} (Fig. S3 *a*, *left*). Consequently, all dimers within the aperture-restricted region are fully photobleached (Fig. S3, *b–d*, *left*). Recovered dimers are either detected as apparent dimers (~90–92%, *black solid line*) or, if two dimers randomly colocalize, as apparent tetramers (~7–9%, *black dashed-dotted line*) (Fig. 3 *a*).

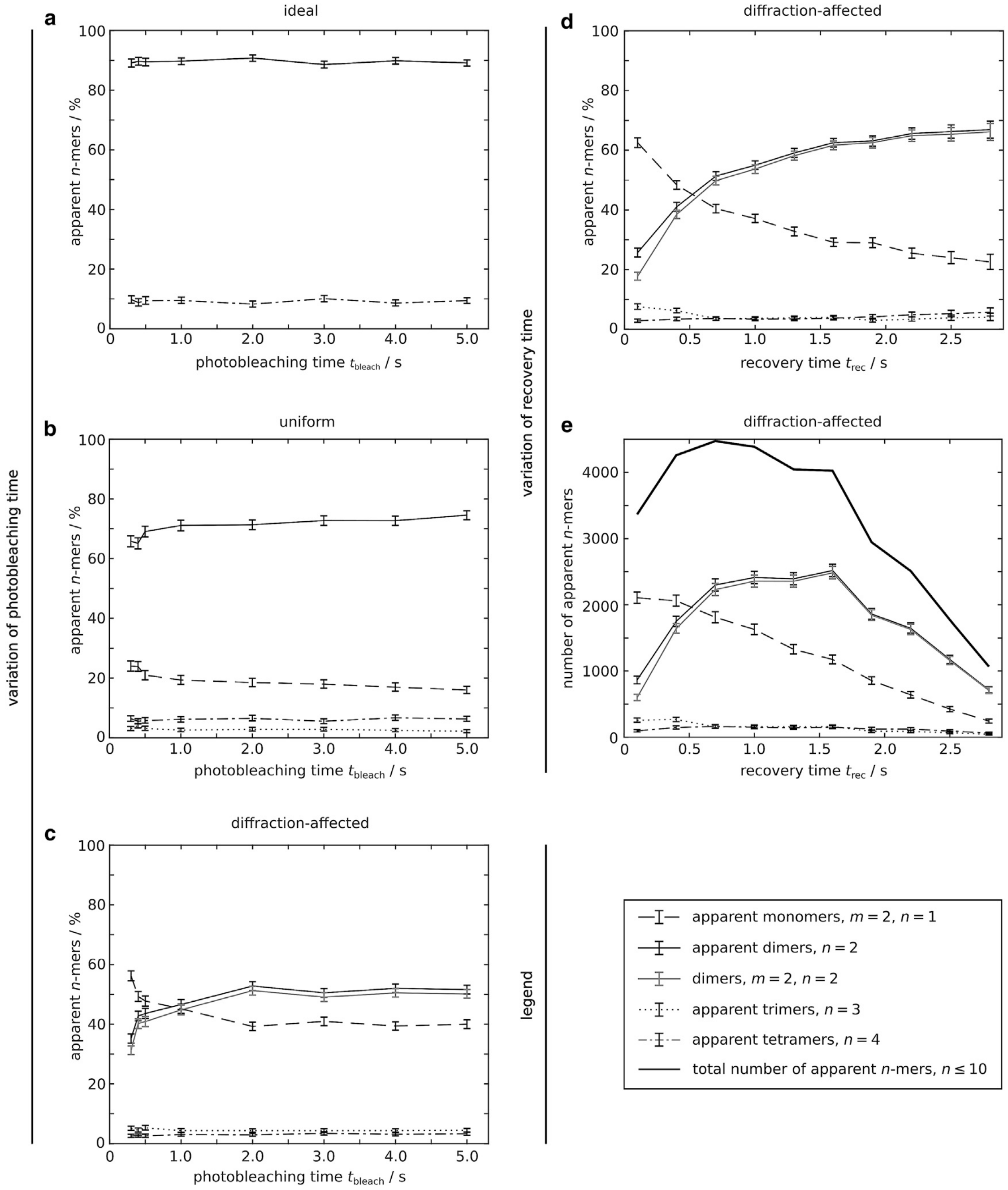

FIGURE 3 The influence of t_{bleach} and t_{rec} on the apparent n-mer fractions was studied. (*a–c*) A purely dimeric population with $D = 0.5\ \mu m^2/s$ was exposed to photobleaching assuming an ideal laser intensity profile (*a*), a uniform laser intensity profile (*b*), and a diffraction-affected laser intensity profile (*c*). The optimal t_{rec} and the according analysis region were determined individually for each t_{bleach}. The gray solid line depicts the mean fraction of dimers excluding apparent dimers due to random colocalizations. In the uniform case the mean fraction of apparent dimers due to random colocalizations is <0.6% and is omitted from the figure. For all three cases the mean apparent n-mer fractions for $n > 4$ are <0.7% (not shown). (*d* and *e*) The influence of t_{rec} on the apparent n-mer fractions was studied. A pure dimeric population with $D = 0.5\ \mu m^2/s$ was exposed to photobleaching for $t_{bleach} = 4$ s, assuming a diffraction-affected laser intensity profile. t_{rec} was varied between 0.1 and 2.8 s and the according analysis region was determined individually for each t_{rec}. (*d*) With increasing t_{rec} the mean fraction of apparent dimers increases, whereas the mean fraction of apparent monomers decreases. The mean apparent n-mer fractions for $n > 4$ are <0.8% (not shown). (*e*) The total number of apparent n-mers within the analysis region first increases with increasing t_{rec} and decreases for $t_{rec} \geq 0.7$ s. Shown are the mean apparent n-mer fractions (*a–d*) and the number of apparent n-mers (sum over 1000 simulation runs) (*e*) and the error bar (95% bootstrapping confidence interval) from 1000 independent simulations. The lines are a guide to the eye.

In the case of a uniform laser intensity profile, p_{bleach} follows Eq. 3 for each time step that each molecule resides within the aperture-restricted region during the photobleaching process (Fig. S3 *a*, *middle*). In this scenario, diffraction effects of the laser at the aperture edges are not considered to show the mere influence of the molecules' movement during illumination. Some dimers leave and enter the aperture-restricted region during photobleaching and are only illuminated for a fraction of t_{bleach} ($t_{ill} \leq t_{bleach}$). Consequently, there is a substantial concentration of partially photobleached dimers near the aperture edges (Fig. S3, *c* and *d*, *middle*), which are detected as apparent monomers after recovery (Fig. 3 *b*, *black dashed line*). Partial photobleaching is even more pronounced in the case of a diffraction-affected laser intensity profile (Fig. 3 *c*) due to the intensity decay and the reduced local p_{bleach} at the aperture edges (Fig. S3, *a*, *c*, and *d*, *right*). Apparent trimers (Fig. 3, *b* and *c*, *black dotted line*) and apparent tetramers (Fig. 3, *b* and *c*, *black dashed-dotted line*) are recorded due to random colocalizations. If two apparent monomers colocalize after t_{rec} they are detected as one apparent dimer.

In the case of the uniform and the diffraction-affected laser intensity profile, for short t_{bleach} some dimers in the center of the aperture-restricted region are not fully photobleached, as exemplified for $t_{bleach} = 0.01$ s and for $t_{bleach} = 0.25$ s (Fig. S3, *b* and *c*, *middle* and *right*). With increasing t_{bleach}, an increasing number of dimers around the aperture-restricted region are fully photobleached, as illustrated in Fig. S3 *d*. For scenarios with longer t_{bleach} the number of (apparent) dimers within the analysis region increases with increasing t_{bleach} (Fig. S7, *a* and *b*).

In the case of the uniform laser intensity profile, for $t_{bleach} =$ 0.3 s, 66% apparent dimers (~65% dimers), ~24% apparent monomers, ~3% apparent trimers, and ~6% apparent tetramers are detected (Fig. 3 *b*; see Table S1 for all *n*-mer fractions). For $t_{bleach} = 4$ s, the fraction of apparent dimers increases to ~73% and does not change substantially with further increasing t_{bleach}. In the case of the diffraction-affected laser intensity profile for $t_{bleach} = 0.3$ s, 35% apparent dimers (~31% dimers) and ~56% apparent monomers are detected (Fig. 3 *c*; see Table S2 for all *n*-mer fractions). For $t_{bleach} =$ 4 s the apparent dimer fraction increases to ~52%.

For all following simulations, a central quadratic aperture-restricted region was photobleached for $t_{bleach} = 4$ s assuming a diffraction-affected laser intensity profile, since for both dimers and tetramers as well as for different diffusion coefficients apparent *n*-mer fractions do not change substantially with higher values of t_{bleach} (Figs. 3 *c* and S8, *a*–*c*).

Influence of recovery time on the apparent dimer fraction

We investigated the influence of t_{rec} on the outcome of a TOCCSL experiment, assuming a purely dimeric population of molecules ($m = 2$) with $D = 0.5$ μm^2/s. For t_{rec} between 0.1 and 2.8 s, in silico TOCCSL experiments were performed. Fig. 3 *d* displays each fraction and Fig. 3 *e* the number of apparent *n*-mers after recovery.

For long t_{rec}, predominantly nonphotobleached dimers from outside the illuminated area reach the analysis region, resulting in a high fraction of dimers (*gray solid line*). However, long recovery times correspond to rather small analysis areas and, hence, low numbers of analyzed molecules, as exemplified for $t_{rec} = 2.8$ s: a total number of 1067 apparent *n*-mers is detected in 1000 TOCCSL simulations, out of which ~67% are apparent dimers (~66% dimers) and ~23% are apparent monomers (see Table S3 for all *n*-mer fractions). When decreasing t_{rec} to 0.7 s, due to the larger analysis region the number of apparent *n*-mers increases to a maximum of 4474. However, an increased number of apparent monomers (*black dashed line*) are detected due to partially photobleached dimers residing close to the aperture edges after t_{bleach} (Fig. S3 *d*, *right*; Table S3), yielding a decreased apparent dimer fraction of ~51%. When further decreasing t_{rec} to values below 0.7 s, both the fraction of apparent dimers and the number of apparent *n*-mers decrease.

In a different set of simulations, a central circular aperture-restricted region was photobleached to investigate influences of the aperture shape on the outcome of a TOCCSL experiment. For both, the circular (Fig. S9) and the rectangular aperture (Fig. 3, *d* and *e*), the mean fractions of apparent *n*-mers were found to be similar.

Influence of mobility on the apparent dimer fraction

We next investigated the influence of D on the outcome of a TOCCSL experiment assuming a purely dimeric population of molecules ($m = 2$). In general, with increasing mobility, an increasing number of dimers leave and enter the aperture-restricted region during t_{bleach}, resulting in increased partial photobleaching and, hence, an increased fraction of apparent monomers (Fig. 4 *a*, *black dashed line*). At lower values of D, less dimers are partially photobleached and more nonphotobleached dimers from outside the illuminated area reach the analysis region, resulting in an increased apparent dimer fraction (*black solid line*). For slowly moving dimers with $D = 0.02$ μm^2/s, ~68% apparent dimers and ~20% apparent monomers are detected compared with ~52% apparent dimers and ~39% apparent monomers for $D = 0.5$ μm^2/s (see Table S4 for all analysis regions and optimal t_{rec}, and Table S5 for all *n*-mer fractions).

Influence of mobility differences on the apparent dimer fraction

If two different oligomeric species exhibit a difference in their mobility, the detection of fast-moving oligomers is favored as they re-populate the analysis region within a shorter amount of time. We investigated the influence of

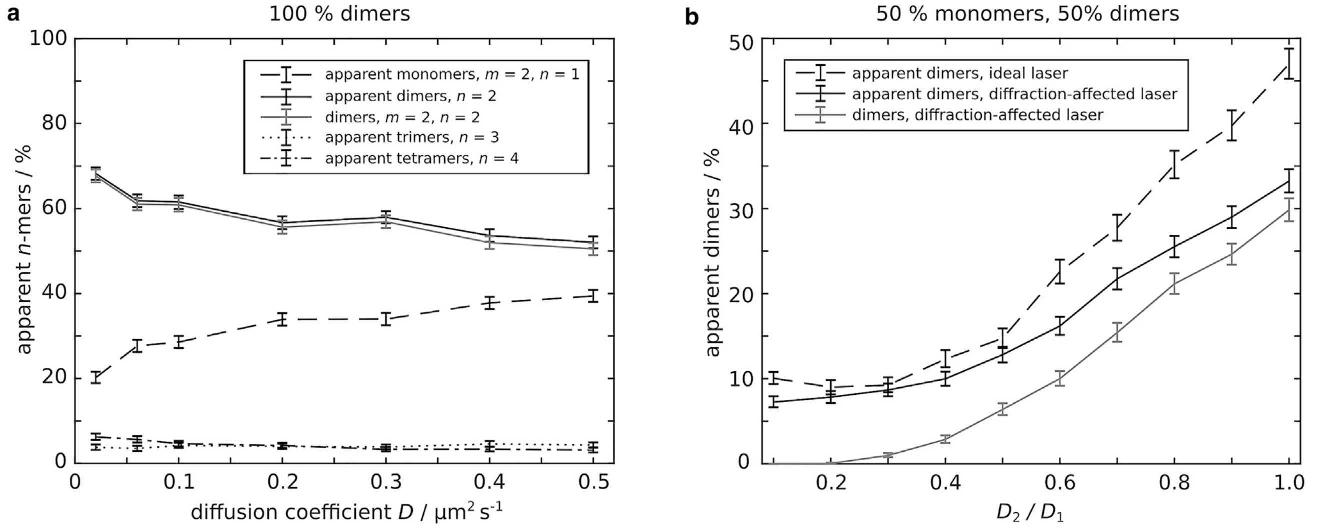

FIGURE 4 The influence of D and the influence of mobility differences between monomers and dimers on the apparent n-mer fractions were studied. (*a*) A purely dimeric population was exposed to photobleaching for $t_{bleach} = 4$ s assuming a diffraction-affected laser intensity profile. The optimal t_{rec} and the according analysis region were determined individually for each D. Apparent n-mer fractions for $n > 4$ were <0.9% (not shown). The apparent n-mer fraction for $D = 0.5$ µm^2/s was taken from Fig. 3 *c*. (*b*) The influence of mobility differences between monomers and dimers on the (apparent) dimer fraction was studied. The monomeric diffusion coefficient D_1 was kept constant at 0.5 µm^2/s while D_2 was varied. A 1:1 distribution of monomers and dimers was exposed to photobleaching for $t_{bleach} = 4$ s assuming an ideal laser intensity profile (*black dashed line*) and a diffraction-affected laser intensity profile (*black solid line*). The gray line depicts the mean fraction of dimers excluding apparent dimers due to random colocalizations. Apparent n-mers due to random colocalizations for $n > 2$ and apparent monomers are not shown. The optimal t_{rec} and the according analysis region were determined individually for each ratio D_2/D_1. Shown are the mean apparent n-mer fractions and error bars (95% bootstrapping confidence interval) from 1000 independent simulations. The lines are a guide to the eye.

mobility differences between different kinds of oligomers on the outcome of a TOCCSL experiment, assuming a 1:1 distribution of monomers and dimers with different diffusion coefficients D_1 and D_2. For all experiments, the diffusion coefficient for monomers was kept constant at $D_1 =$ 0.5 µm^2/s, while the ratio of diffusion coefficients D_2/D_1 was varied from 0.1 to 1. A central region was photobleached assuming an ideal and a diffraction-affected laser intensity profile. Fig. 4 *b* displays the (apparent) dimer fraction after recovery.

The ideal scenario (*black dashed line*) shows the mere influence of mobility differences, as nearly all dimers are detectable after recovery (see influence of photobleaching time on the apparent dimer fraction). In the case of D_2/$D_1 = 1$ ~47% apparent dimers are detected, whereas ~3% of dimers are detected as higher-order n-mers due to random colocalizations (Table S6). With decreasing D_2, t_{rec} increases and an increasing number of monomers reach the analysis region. Consequently, a lower fraction of apparent dimers is detected. For $D_2/D_1 = 0.3$ ~9% apparent dimers are detected.

The dependence of the apparent dimer fraction on the ratio D_2/D_1 is even more pronounced in the case of a diffraction-affected laser intensity profile. Here, partial photobleaching additionally prevents the detection of apparent dimers (*black solid line*). For $D_2/D_1 = 1$ ~33%, apparent dimers (~30% dimers) are detected (Table S6). For $D_2/D_1 = 0.3$ the fraction of apparent dimers drops to ~9% (~1% dimers). It is instructive to plot the fraction of apparent dimers, but excluding those events that are due to randomly colocalized molecules (*gray solid line*) as for $D_2/D_1 = 0.3$ predominantly and for $D_2/D_1 \leq 0.2$ exclusively apparent dimers due to random colocalizations are detected. The counteracting influences of partial photobleaching and random colocalizations are given by the respective areas below the curves. The predominant effect, however, is the decreasing number of dimers in the analysis region with increasing mobility differences between monomers and dimers.

Influence of partial photobleaching and random colocalizations on the apparent tetramer fraction

Finally, we investigated the influence of partial photobleaching and random colocalizations on the detection of higher-order oligomers in in silico TOCCSL experiments. We assumed a purely tetrameric population of molecules ($m = 4$) with either a diffusion coefficient of $D = 0.02$ µm^2/s, $D = 0.1$ µm^2/s, or $D = 0.5$ µm^2/s. In addition, we included two scenarios assuming an equal mixture of two tetrameric populations with diffusion coefficients $D_1 =$ 0.02 µm^2/s and $D_2 = 0.1$ µm^2/s or $D_1 = 0.1$ µm^2/s and $D_2 = 0.5$ µm^2/s. To photobleach all tetramers in the center of the aperture-restricted region with a probability of virtually 100%, a t_{bleach} of 4 s was sufficient (Figs. S8 *c* and S10).

Fig. 5 displays each fraction of apparent n-mers after recovery. As in the case of dimers (see influence of mobility

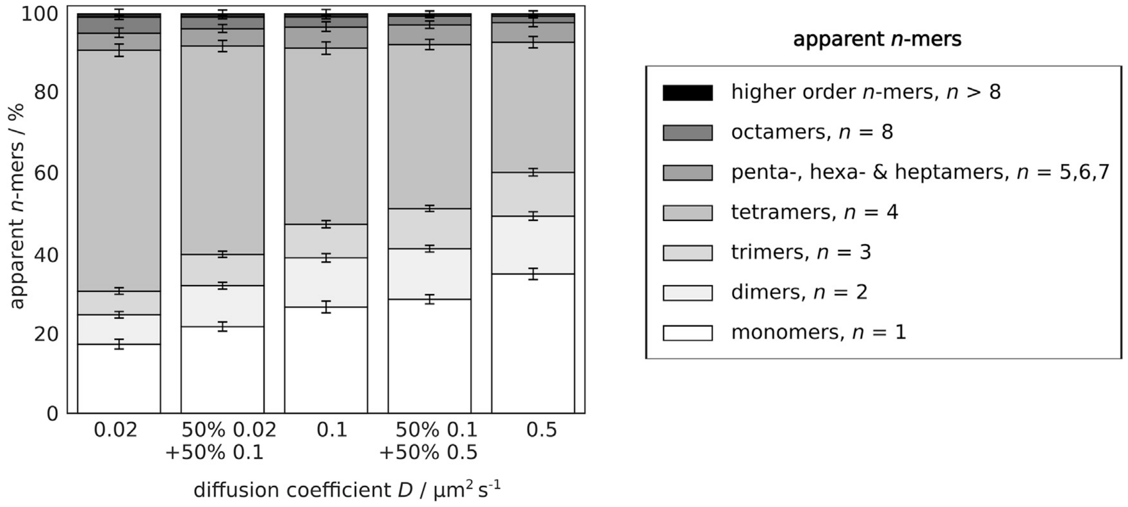

FIGURE 5 The influence of partial photobleaching and random colocalizations on the apparent *n*-mer fractions of tetrameric populations was studied. We studied purely tetrameric populations of molecules ($m = 4$) with a diffusion coefficient of either $D = 0.02$ μm^2/s, $D = 0.1$ μm^2/s, or $D = 0.5$ μm^2/s (columns 1, 3, and 5). In addition, we included two scenarios assuming an equal mixture of two tetrameric populations with diffusion coefficients $D_1 = 0.02$ μm^2/s and $D_2 = 0.1$ μm^2/s (column 2) or $D_1 = 0.1$ μm^2/s and $D_2 = 0.5$ μm^2/s (column 4). Molecules were exposed to photobleaching for $t_{\mathrm{bleach}} = 4$ s, assuming a diffraction-affected laser intensity profile. The optimal t_{rec} and the according analysis region were determined for each D. Shown are the mean apparent *n*-mer fractions and error bars (95% bootstrapping confidence interval) from 1000 independent simulations.

on the apparent dimer fraction) assuming fast mobility of $D = 0.5$ μm^2/s, a high number of tetramers leave and enter the aperture-restricted area during photobleaching. Consequently, a high number of partially photobleached tetramers from the aperture edges—predominantly apparent monomers—reach the analysis region. In this scenario ~33% apparent tetramers (~32% tetramers) are detected (see Table S7 for all *n*-mer fractions). With decreasing D, less tetramers undergo partial photobleaching and an increasing number of nonphotobleached tetramers reach the analysis region. For $D = 0.1$ μm^2/s ~44% tetramers (~44% tetramers) and for slowly moving tetramers with $D = 0.02$ μm^2/s ~60% apparent tetramers (~60% tetramers) are detected (Table S7). For the mixed population with $D_1 = 0.02$ μm^2/s and $D_2 = 0.1$ μm^2/s ~52% apparent tetramers (~52% tetramers) and for the mixed population with $D_1 = 0.1$ μm^2/s and $D_2 = 0.5$ μm^2/s ~41% apparent tetramers (~40% tetramers) are detected (Table S7).

The effect of the recovery time on the detection of a purely tetrameric population was studied in a separate set of TOCCSL simulations for $D = 0.5$ μm^2/s (Fig. S8, *d* and *e*). As expected, for short t_{rec} a high number of apparent monomers are detected, which reside close to the aperture edges after photobleaching. With increasing t_{rec}, an increasing number of nonphotobleached tetramers from outside the illuminated area reach the analysis region, thus increasing the fraction of apparent tetramers.

DISCUSSION

To date, TOCCSL has been successfully applied to study the homo-oligomeric composition of various plasma membrane protein and lipid complexes. For transmembrane proteins in live cells, the degree of measured oligomerization ranged from a pure monomer population of the T cell receptor/CD3 complex (23), to a mixed population of monomers and dimers for the dopamine transporter (24), a mixed population of monomers and PIP2-stabilized higher-order oligomers for the serotonin transporter (25,26), up to tetramers described for the subunit composition of Orai1 (27). Monomeric green fluorescent protein linked via a glycosylphosphatidylinositol anchor (mGFP-GPI) to the plasma membrane of CHO cells was found to form homo-associates, while the glycolipid GM1 was confined to cholesterol-dependent nanodomains hosting up to four GM1 molecules on the surface of Jurkat T cells (19). While the quantification of oligomerization could be routinely performed for the examples listed, the characterization of ErbB3 homo- and hetero-oligomerization was challenged by the dependence of the diffusion coefficients on degree and type of oligomerization, yielding an overrepresentation of monomeric ErbB3 and ErbB3 dimers close to the detection limit before correction (28).

To quantitatively characterize the influence of different diffusion coefficients and experimental parameters on the oligomerization result gained in a TOCCSL measurement, we performed in silico TOCCSL experiments. For simplicity and easy interpretation of the results we utilized Monte Carlo-based simulations of diffusing dimers exposed to photobleaching followed by a recovery step and quantification of oligomerization (Figs. 1, 2, and S6). We could identify the diffraction-affected laser intensity profile as a major source of generating partially photobleached dimers, which amounts to a decreased fraction of apparent dimers.

Partial photobleaching of dimers is caused by two effects at the edges of the aperture-restricted region: 1) diffusion of dimers in and out of the illuminated region during the photobleaching pulse and 2) an illumination intensity decay yielding a reduced photobleaching probability. In the following we provide an overview of parameters and how they influence the fraction of apparent dimers:

a. Photobleaching time (Figs. 3, *a–c*, and S8, *a–c*): t_{bleach} must be sufficiently long to photobleach all dimers in the central region. Interestingly, long photobleaching times were found to be unproblematic or even beneficial to detect higher dimer fractions for all simulated laser intensity profiles. However, for the parameters simulated ($D = 0.5$ $\mu m^2/s$) only about 52% of apparent dimers could be detected in a real case scenario. Note that even for an ideal photobleaching process the dimer fraction is reduced due to the detection of apparent tetramers caused by random colocalizations.
b. Diffusion coefficient of dimers: larger diffusion coefficients result in a higher number of partially photobleached dimers mainly caused by the aforementioned effects at the aperture edges. While the apparent dimer fraction yielded only ~52% for $D = 0.5$ $\mu m^2/s$, it increased to ~68% for $D = 0.02$ $\mu m^2/s$ (Fig. 4 *a*). A similar dependency could be shown for tetramers (Fig. 5).
c. Recovery time: t_{rec} was found to represent an important parameter affecting the fraction and number of detected dimers (Figs. 3, *d* and *e*, and S8, *d* and *e*). Long recovery times allow for more nonphotobleached dimers to diffuse from outside the photobleached area into the analysis region and hence result in higher dimer fractions. However, too long recovery times result in a high density of molecules. In consequence, the analysis area, in which the probability for random colocalizations is below the selected threshold, gets rather small. In our simulations we selected the recovery time based on the maximum number of molecules accepted for analysis (Figs. 2 and S6), thereby maximizing the number of signals for reliable analysis. Plotting the total number of all molecules within the analysis region versus the respective apparent *n*-mer fraction (see Fig. S11 for an example of dimers) shows the trade-off of the chosen selection: while at the maximum number of analyzed molecules an apparent dimer fraction of ~51% is determined, the maximal apparent dimer fraction of ~67% is determined from a substantially lower number of analyzed molecules.
d. Different diffusion coefficients of monomers and dimers: as shown in the case of ErbB3 experimentally (28), the diffusion coefficient can also depend on the degree of oligomerization and/or activation state, yielding a bias toward the fraction of the fast-diffusing species. To address this effect in more detail, we simulated a mixed population of 50% monomers and 50% dimers and determined the fraction of detected dimers upon decreasing the diffusion coefficient of the dimer population while keeping the diffusion coefficient of monomers constant (Fig. 4 *b*). Already an approximately threefold reduced diffusion coefficient diminished the dimer fraction to only ~1%. This effect is counteracted by random colocalizations caused by the presence of fast-diffusing monomers within the analysis region, leading to a fraction of apparent dimers of ~9%. Hence, higher differences in diffusion coefficients with dimer fractions below 1% demand for detecting rare dimerization events, e.g., by using two-color TOCCSL (20).

Taken together, Monte Carlo-based simulations allowed for the identification and quantification of error sources in detecting molecular dimers and tetramers in TOCCSL experiments. It is important to note that, in a real microscopy-based TOCCSL experiment, additional error sources associated with single-molecule detection (29–32) are present (e.g., noise in determining the accurate single-molecule brightness values and noise in assigning fluorescent/oligomeric states) and will additionally influence the final outcome of a TOCCSL experiment. Individual error sources can be determined and considered separately for correcting oligomer fractions.

The gained knowledge from in silico TOCCSL can further be utilized for:

a. The correction of apparent *n*-mer fractions gained in microscopy-based TOCCSL experiments. Experimental shortcomings described before yield a decreased apparent dimer fraction compared with the ground truth. In silico TOCCSL can be utilized to correct apparent *n*-mer fractions, yielding a better estimate of the true dimer fraction. A detailed characterization of the experimental system to determine a priori knowledge of surface densities, diffusion coefficients, photobleaching parameters, and labeling efficiencies is the requirement for conducting in silico TOCCSL based on the same parameters used for microscopy experiments, namely photobleaching, recovery time, and aperture size (see Figures S6 for all parameters). After selecting an appropriate oligomerization model for the molecule under investigation, e.g., based on the crystal structure or other experimental evidence, the ground truth oligomeric fractions are varied in TOCCSL simulations as shown in the flow chart (Figures S6). As soon as the apparent *n*-mer fractions match the results from the microscopy experiment the true oligomeric fractions can be extracted. Note that the analysis needs to be conducted the same way for both the in silico- and microscopy-based TOCCSL experiments. For simple models, i.e., the existence of only monomers and dimers, the true oligomeric fractions could be determined as shown in (29) using this reverse approach.

b. Finding optimal parameters for microscopy-based TOCCSL experiments. Similar to (a), but simulations are performed before the actual microscopy experiment. By varying all relevant parameters, the optimal recovery time is calculated based on the allowed probability of random colocalizations. Furthermore, the choice of photobleaching time and aperture size can be optimized. Using parameters gained from simulations ensures the most sensitive detection of accurate oligomeric states in microscopy experiments. An additional in silico TOCCSL simulation as outlined in (a) will allow for correcting the microscopy-based results and potential refinement of suitable TOCCSL parameters as part of an iterative approach.

Finally, the presented simulations can easily be extended to accommodate more complex oligomerization models involving different oligomeric fractions. More dynamic models assuming not only the existence of stable oligomers could routinely be included. In general, dynamic oligomerization can be quantified by using TOCCSL in two different types of experiments: 1) if association times (t_{off}) are at the order of several seconds to minutes (i.e., t_{off} considerably exceeds the time needed for one TOCCSL experiment), one can repeat TOCCSL experiments on the very same cells to estimate association times. In this case, the multiple photobleaching pulses applied to the same cell will yield decreasing fractions of higher-order apparent n-mers due to the exchange of photobleached and nonphotobleached subunits. Depending on the chosen time interval between TOCCSL runs and the ratio of photobleached cell area versus total cell area, dynamic parameters can be estimated. This strategy was recently used to address whether subunits of SERT (25), DAT (24), and NET (33) oligomers are exchanged on the time scale of minutes. 2) If t_{off} is comparable with the time needed for one TOCCSL experiment, variation of the recovery time will yield different apparent n-mer fractions, which allows for estimating dynamic parameters. Based on in silico TOCCSL, optimal experimental timings can be chosen such that the bias toward lower apparent n-mer fractions is minimized and correct oligomeric fractions can be calculated. Importantly, for very weak interactions ($t_{off} << t_{rec}$), higher-order oligomers will be underrepresented due to the fast exchange of abundant photobleached subunits with nonphotobleached subunits during the recovery process.

Also, nonideal labeling conditions like degree of labeling, maturation grade of fluorescent proteins, etc.—which have been neglected in our study to keep interpretation of results simple—can be embedded in in silico TOCCSL. For the same reason of simplicity, we did not vary the bleaching probability or adapt the size of the observed sample to simulate other specimen types. Our approach allows for accounting different methods to photobleach a defined area, e.g., TIR, non-TIR, or a scanned focused laser beam; for this, the photobleaching probability can be adapted in our algorithm accordingly. For all simulations we assumed mammalian cells and varied the size between $10.5 \cdot 10.5$ and $28 \cdot 28$ μm^2. Importantly, cell size and photobleached area can be easily adapted to perform simulations for other systems, e.g., to mimic smaller organisms like bacteria.

SUPPORTING MATERIAL

Supporting material can be found online at https://doi.org/10.1016/j.bpj.2023.04.021.

AUTHOR CONTRIBUTIONS

M.B. and G.J.S. conceived the project. C.B. and D.K. designed the simulations and analyzed the simulated data. D.K. measured and analyzed photobleaching curves and laser profiles. C.B., M.B., and G.J.S. wrote the manuscript.

ACKNOWLEDGMENTS

This work has been supported by the Austrian Science Fund (FWF) project numbers I 5056-B (to G.J.S.), I 4662-B and I 4740 (to M.B.), and by the doctoral college Biointerface (TU Wien, Vienna, Austria).

DECLARATION OF INTERESTS

The authors declare no competing interests.

REFERENCES

1. Torreno-Pina, J. A., C. Manzo, and M. F. Garcia-Parajo. 2016. Uncovering homo-and hetero-interactions on the cell membrane using single particle tracking approaches. *J. Phys. D Appl. Phys.* 49, 104002. https://doi.org/10.1088/0022-3727/49/10/104002.
2. Wilmes, S., M. Hafer, ..., J. Piehler. 2020. Mechanism of homodimeric cytokine receptor activation and dysregulation by oncogenic mutations. *Science.* 367:643–652. https://doi.org/10.1126/science.aaw3242.
3. Sotolongo Bellón, J., O. Birkholz, ..., J. Piehler. 2022. Four-color single-molecule imaging with engineered tags resolves the molecular architecture of signaling complexes in the plasma membrane. *Cell Rep. Methods.* 2, 100165. https://doi.org/10.1016/j.crmeth.2022.100165.
4. Beck, M., A. Schmidt, ..., R. Aebersold. 2011. The quantitative proteome of a human cell line. *Mol. Syst. Biol.* 7:549. https://doi.org/10.1038/msb.2011.82.
5. Douglass, A. D., and R. D. Vale. 2005. Single-molecule microscopy reveals plasma membrane microdomains created by protein-protein networks that exclude or trap signaling molecules in T cells. *Cell.* 121:937–950. https://doi.org/10.1016/j.cell.2005.04.009.
6. Belyy, V., S. M. Shih, ..., A. Yildiz. 2017. PhotoGate microscopy to track single molecules in crowded environments. *Nat. Commun.* 8, 13978. https://doi.org/10.1038/ncomms13978.
7. Vrljic, M., S. Y. Nishimura, ..., H. M. McConnell. 2002. Translational diffusion of individual class II MHC membrane proteins in cells. *Biophys. J.* 83:2681–2692. https://doi.org/10.1016/S0006-3495(02)75277-6.
8. Manley, S., J. M. Gillette, ..., J. Lippincott-Schwartz. 2008. High-density mapping of single-molecule trajectories with photoactivated localization microscopy. *Nat. Methods.* 5:155–157. https://doi.org/10.1038/nmeth.1176.

9. Rust, M. J., M. Bates, and X. Zhuang. 2006. Sub-diffraction-limit imaging by stochastic optical reconstruction microscopy (STORM). *Nat. Methods.* 3:793–795. https://doi.org/10.1038/nmeth929.

10. Heilemann, M., S. van de Linde, …, M. Sauer. 2008. Subdiffraction-resolution fluorescence imaging with conventional fluorescent probes. *Angew. Chem. Int. Ed. Engl.* 47:6172–6176. https://doi.org/10.1002/anie.200802376.

11. Betzig, E., G. H. Patterson, …, H. F. Hess. 2006. Imaging intracellular fluorescent proteins at nanometer resolution. *Science.* 313:1642–1645. https://doi.org/10.1126/science.1127344.

12. Hess, S. T., T. P. K. Girirajan, and M. D. Mason. 2006. Ultra-high resolution imaging by fluorescence photoactivation localization microscopy. *Biophys. J.* 91:4258–4272. https://doi.org/10.1529/biophysj.106.091116.

13. Sengupta, P., T. Jovanovic-Talisman, …, J. Lippincott-Schwartz. 2011. Probing protein heterogeneity in the plasma membrane using PALM and pair correlation analysis. *Nat. Methods.* 8:969–975. https://doi.org/10.1038/nmeth.1704.

14. Jungmann, R., M. S. Avendaño, …, P. Yin. 2016. Quantitative super-resolution imaging with qPAINT. *Nat. Methods.* 13:439–442. https://doi.org/10.1038/nmeth.3804.

15. Honigmann, A., V. Mueller, …, C. Eggeling. 2014. Scanning STED-FCS reveals spatiotemporal heterogeneity of lipid interaction in the plasma membrane of living cells. *Nat. Commun.* 5:5412. https://doi.org/10.1038/ncomms6412.

16. Manzo, C., T. S. van Zanten, and M. F. Garcia-Parajo. 2011. Nanoscale fluorescence correlation spectroscopy on intact living cell membranes with NSOM probes. *Biophys. J.* 100:L8–L10. https://doi.org/10.1016/j.bpj.2010.12.3690.

17. Moertelmaier, M., M. Brameshuber, …, H. Stockinger. 2005. Thinning out clusters while conserving stoichiometry of labeling. *Appl. Phys. Lett.* 87, 263903. https://doi.org/10.1063/1.2158031.

18. Axelrod, D., D. E. Koppel, …, W. W. Webb. 1976. Mobility measurement by analysis of fluorescence photobleaching recovery kinetics. *Biophys. J.* 16:1055–1069. https://doi.org/10.1016/S0006-3495(76)85755-4.

19. Brameshuber, M., J. Weghuber, …, G. J. Schütz. 2010. Imaging of mobile long-lived nanoplatforms in the live cell plasma membrane. *J. Biol. Chem.* 285:41765–41771. https://doi.org/10.1074/jbc.M110.182121.

20. Ruprecht, V., M. Brameshuber, and G. J. Schütz. 2010. Two-color single molecule tracking combined with photobleaching for the detection of rare molecular interactions in fluid biomembranes. *Soft Matter.* 6:568–581. https://doi.org/10.1039/B916734J.

21. Brameshuber, M., and G. J. Schütz. 2012. Detection and quantification of biomolecular association in living cells using single-molecule microscopy. *Methods Enzymol.* 505:159–186. https://doi.org/10.1016/B978-0-12-388448-0.00017-6.

22. Bodner, C., and M. Brameshuber. 2022. Determination of Biomolecular Oligomerization in the Live Cell Plasma Membrane via Single-Molecule Brightness and Co-localization Analysis. Springer Series on Fluorescence. Springer.https://doi.org/10.1007/4243_2022_25.

23. Brameshuber, M., F. Kellner, …, J. B. Huppa. 2018. Monomeric TCRs drive T cell antigen recognition. *Nat. Immunol.* 19:487–496. https://doi.org/10.1038/s41590-018-0092-4.

24. Das, A. K., O. Kudlacek, …, G. J. Schütz. 2019. Dopamine transporter forms stable dimers in the live cell plasma membrane in a phosphatidylinositol 4,5-bisphosphate-independent manner. *J. Biol. Chem.* 294:5632–5642. https://doi.org/10.1074/jbc.RA118.006178.

25. Anderluh, A., E. Klotzsch, …, G. J. Schütz. 2014. Single molecule analysis reveals coexistence of stable serotonin transporter monomers and oligomers in the live cell plasma membrane. *J. Biol. Chem.* 289:4387–4394. https://doi.org/10.1074/jbc.M113.531632.

26. Anderluh, A., T. Hofmaier, …, G. J. Schütz. 2017. Direct PIP2 binding mediates stable oligomer formation of the serotonin transporter. *Nat. Commun.* 8, 14089. https://doi.org/10.1038/ncomms14089.

27. Madl, J., J. Weghuber, …, G. J. Schütz. 2010. Resting state Orai1 diffuses as homotetramer in the plasma membrane of live mammalian cells. *J. Biol. Chem.* 285:41135–41142. https://doi.org/10.1074/jbc.M110.177881.

28. Váradi, T., M. Schneider, …, P. Nagy. 2019. Homo- and heteroassociations drive activation of ErbB3. *Biophys. J.* 117:1935–1947. https://doi.org/10.1016/j.bpj.2019.10.001.

29. Ober, R. J., S. Ram, and E. S. Ward. 2004. Localization accuracy in single-molecule microscopy. *Biophys. J.* 86:1185–1200. https://doi.org/10.1016/S0006-3495(04)74193-4.

30. Mortensen, K. I., L. S. Churchman, …, H. Flyvbjerg. 2010. Optimized localization analysis for single-molecule tracking and super-resolution microscopy. *Nat. Methods.* 7:377–381. https://doi.org/10.1038/nmeth.1447.

31. Thompson, R. E., D. R. Larson, and W. W. Webb. 2002. Precise nanometer localization analysis for individual fluorescent probes. *Biophys. J.* 82:2775–2783. https://doi.org/10.1016/S0006-3495(02)75618-X.

32. Mutch, S. A., B. S. Fujimoto, …, D. T. Chiu. 2007. Deconvolving single-molecule intensity distributions for quantitative microscopy measurements. *Biophys. J.* 92:2926–2943. https://doi.org/10.1529/biophysj.106.101428.

33. Luethi, D., J. Maier, …, H. H. Sitte. 2022. Phosphatidylinositol 4,5-bisphosphate (PIP_2) facilitates norepinephrine transporter dimerization and modulates substrate efflux. *Commun. Biol.* 5:1259. https://doi.org/10.1038/s42003-022-04210-1.

Biophysical Journal, Volume *122*

Supplemental information

Monte Carlo simulations for the evaluation of oligomerization data in TOCCSL experiments

Clara Bodner, Dominik Kiesenhofer, Gerhard J. Schütz, and Mario Brameshuber

Supplemental figures

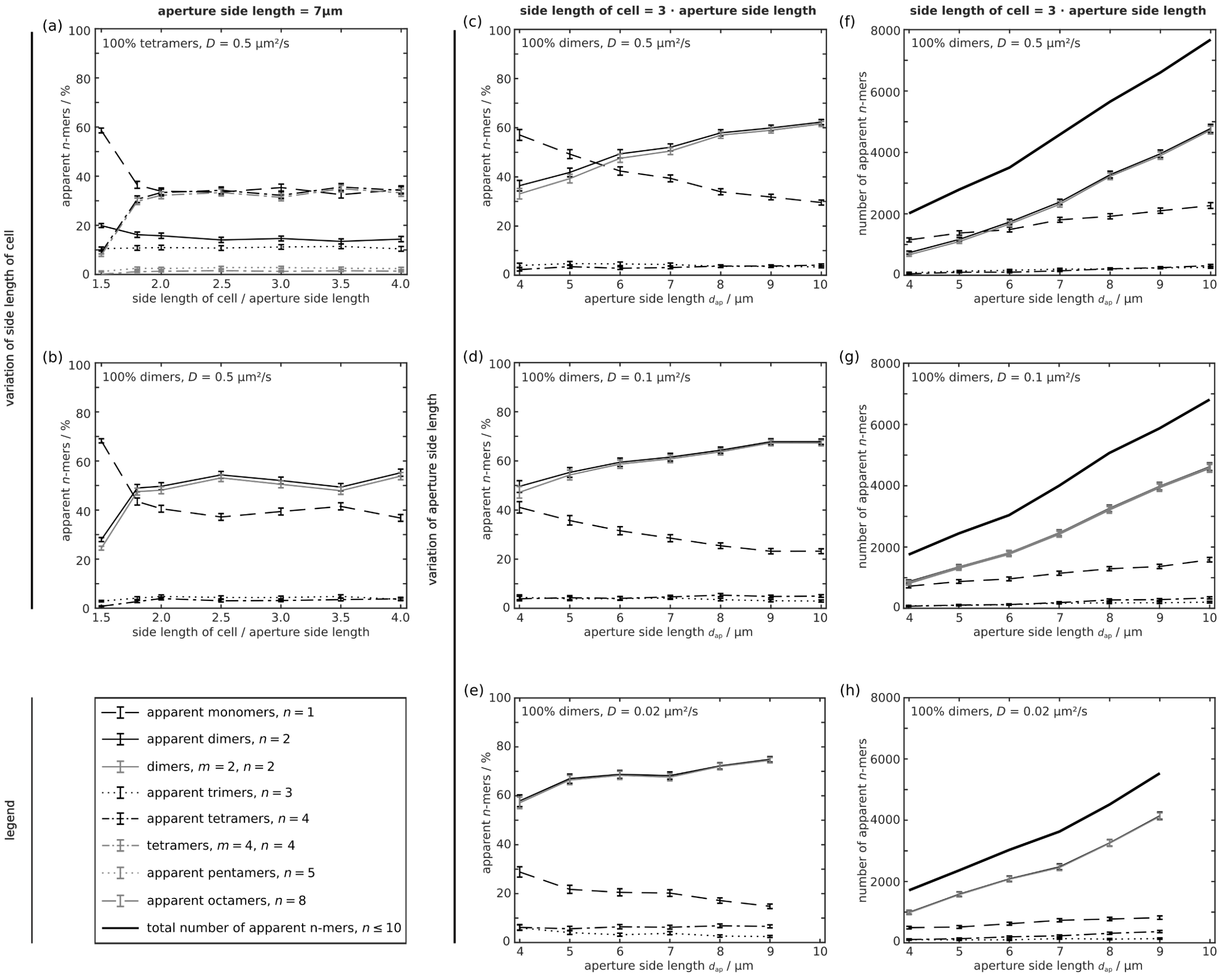

Figure S1 The influence of the simulated cell area (assuming an aperture side length of $d_{ap} = 7$ µm) (a, b) and the influence of the aperture side length d_{ap} (c-h, assuming the side length of the cell to be $3 \cdot d_{ap}$) on the apparent *n*-mer fractions were studied. A purely tetrameric population with $D = 0.5$ µm²/s (a) and purely dimeric populations with $D = 0.5$ µm²/s (b, c, f), $D = 0.1$ µm²/s (d, g) or $D = 0.02$ µm²/s (e, h) were exposed to photobleaching for $t_{bleach} = 4$ s assuming a diffraction-affected laser intensity profile with an intensity decay outside the aperture-restricted region reaching 0 after $d_{edge} = 1$ µm. The optimal t_{rec} and the according analysis region were determined individually for each cell area and each d_{ap}. The detection of apparent monomers (black dashed line) is caused by partial photobleaching, which reduces the fraction of apparent dimers (black solid line). Apparent trimers (dotted line) and apparent tetramers (dashed-dotted line) are present due to random colocalizations. The gray solid line depicts the fraction of dimers excluding apparent dimers due to random colocalizations. Apparent *n*-mer fractions for $n > 4$ are only shown in (a). (a,b): For cell side lengths smaller than 2 times the aperture side length, depletion of fluorescent molecules has to be taken into account. Due to the small cell size and the small, finite reservoir of molecules, most molecules on the cell area are either fully or partially photobleached during illumination. Hence, the probability for the same oligomer to leave and enter the aperture restricted region multiple times during photobleaching is higher compared to larger cell sizes. The high number of partially photobleached oligomers results in a lower fraction of apparent tetramers (a) and apparent dimers (b), compared to larger cell sizes. (c-h): For increasing d_{ap} the recovery time t_{rec} and the size of the analysis region increase and thus, the number as well as the fraction of apparent dimers increase, whereas the fraction of apparent monomers decreases. Shown are the mean apparent *n*-mer fractions (a-e) and the number of apparent *n*-mers (sum over 1000 simulation runs) (f-h) and the error bar (95 % bootstrapping confidence interval) from 1000 independent simulations. The lines are a guide to the eye.

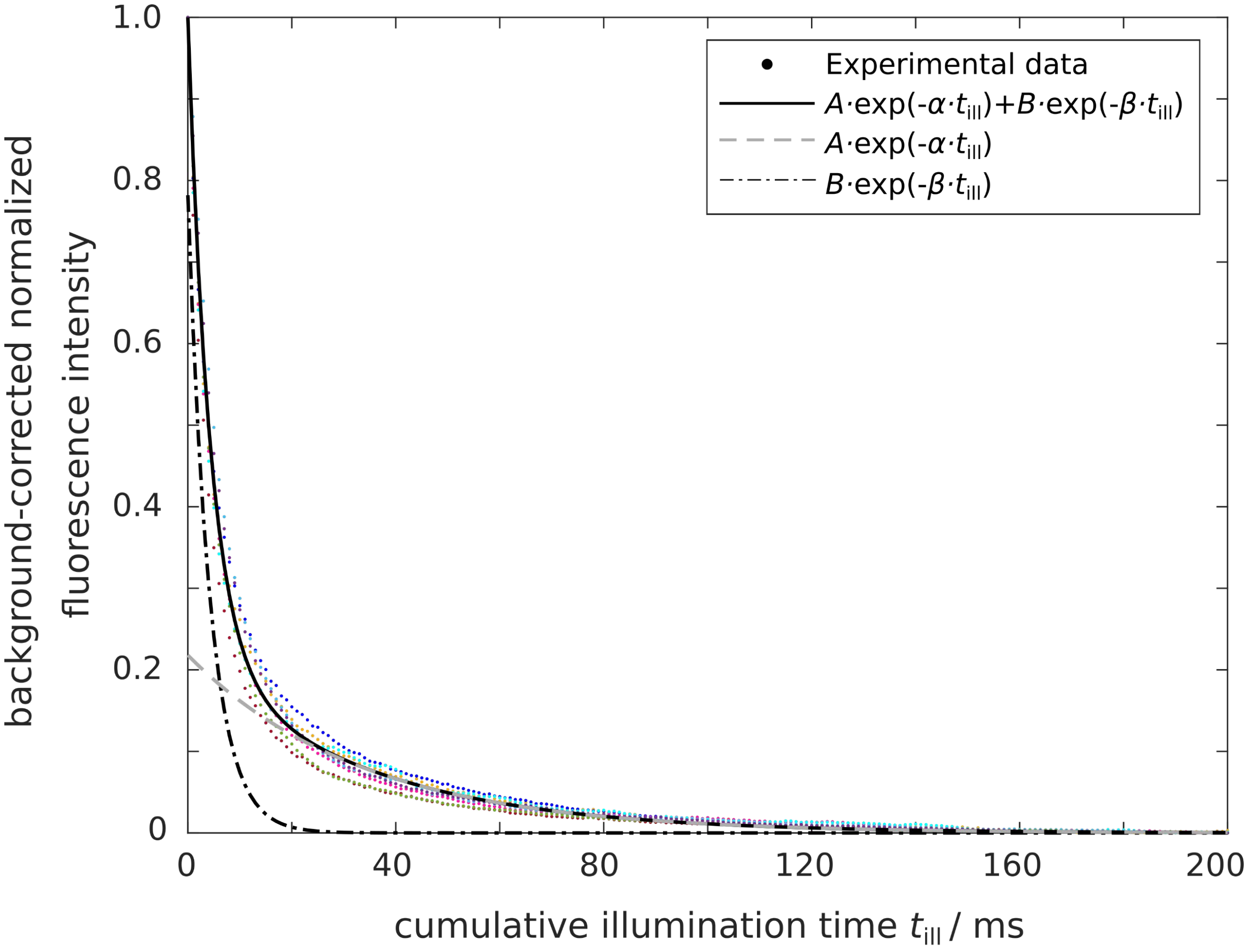

Figure S2 A photobleaching curve was experimentally determined from 8 independent CHO mGFP-GPI cells (colored dots). For each cell 200 images were recorded with an illumination time of $t_{\mathrm{ill}} = 1\,\mathrm{ms}$. Hence, cells were exposed to photobleaching for a time given by the cumulative illumination time of $n \cdot 1\ ms$, with n denoting the frame number. For all 200 images the integrated brightness of the same region of interest (ROI) within the illuminated area was determined, background-corrected, normalized, and fitted via linear least squares by a two-component exponential function. The mean fit parameters derived from 8 cells, $a = 235\,1/\mathrm{s}$, $\beta = 30\,1/\mathrm{s}$, $A = 0.782$ and $B = 0.218$, were used to calculate the photobleaching curve (black line). For the simulations, one photobleaching rate coefficient α or β is assigned to each dye molecule according to its weight A or B.

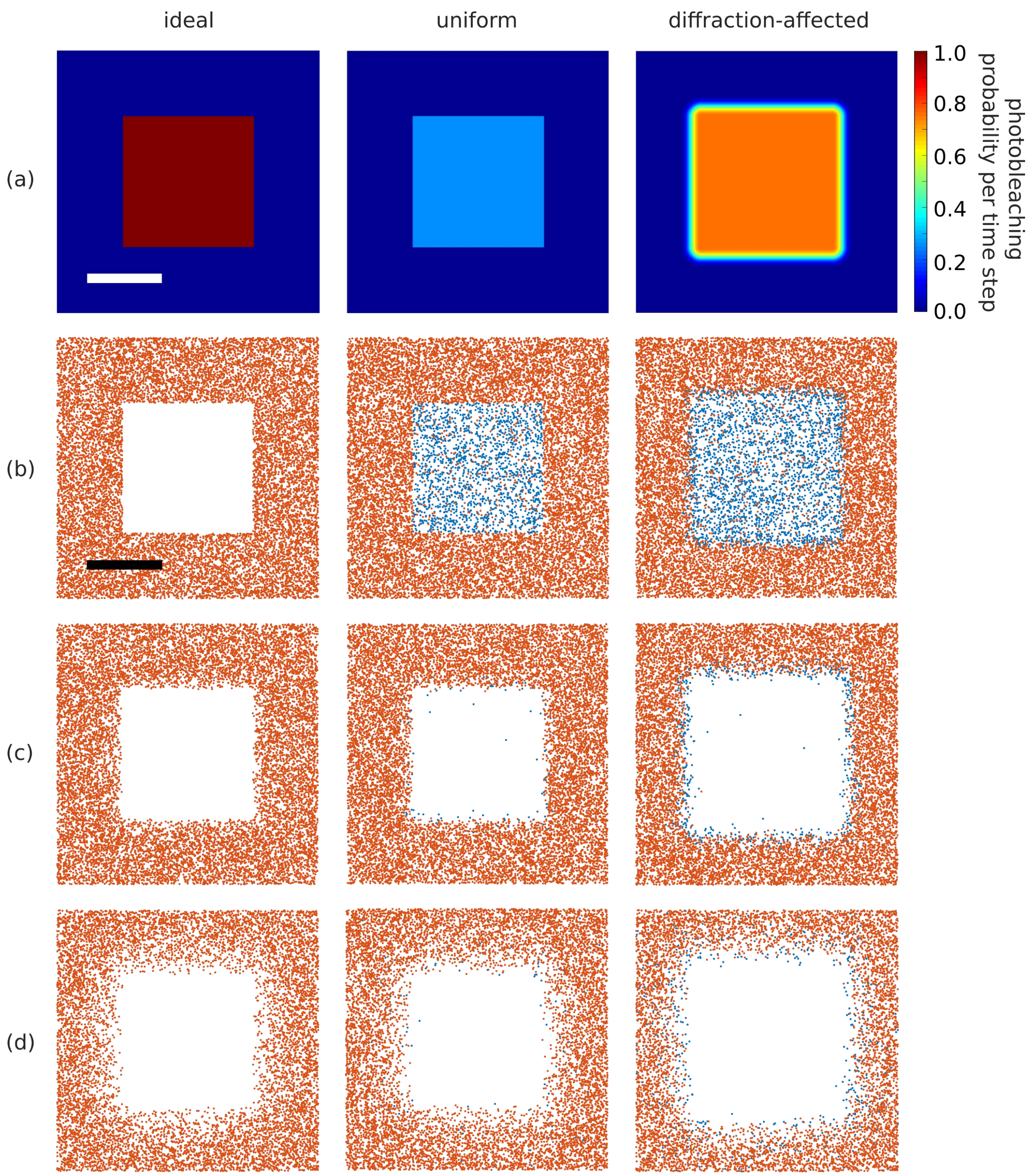

Figure S3 Shown are simulated photobleaching probability profiles for a single simulated time step (a) and exemplary simulated TOCCSL data (b-d), assuming an initial density of 100 dimers/µm² with a diffusion coefficient of $D = 0.5$ µm²/s. The central aperture-restricted region (7·7 µm^2) and the additional edge region of 1 µm (in case of the diffraction-affected laser intensity profile) are photobleached for a single time step ($t_{\text{bleach}} = 0.001$ s) (a, b), for $t_{\text{bleach}} = 0.25$ s (c) and for $t_{\text{bleach}} = 4$ s (d), assuming ideal photobleaching (instantaneous, photobleaching with probability $p_{\text{bleach}} = 1$, sharp aperture edges) (left), uniform photobleaching (time-dependent p_{bleach}, sharp aperture edges) (middle) and diffraction-affected photobleaching (time- and intensity-dependent p_{bleach}, intensity decay at the aperture edges) (right). p_{bleach} outside the illuminated area is 0. Dimers are shown in red, apparent monomers in blue. Scale bar 4 µm.

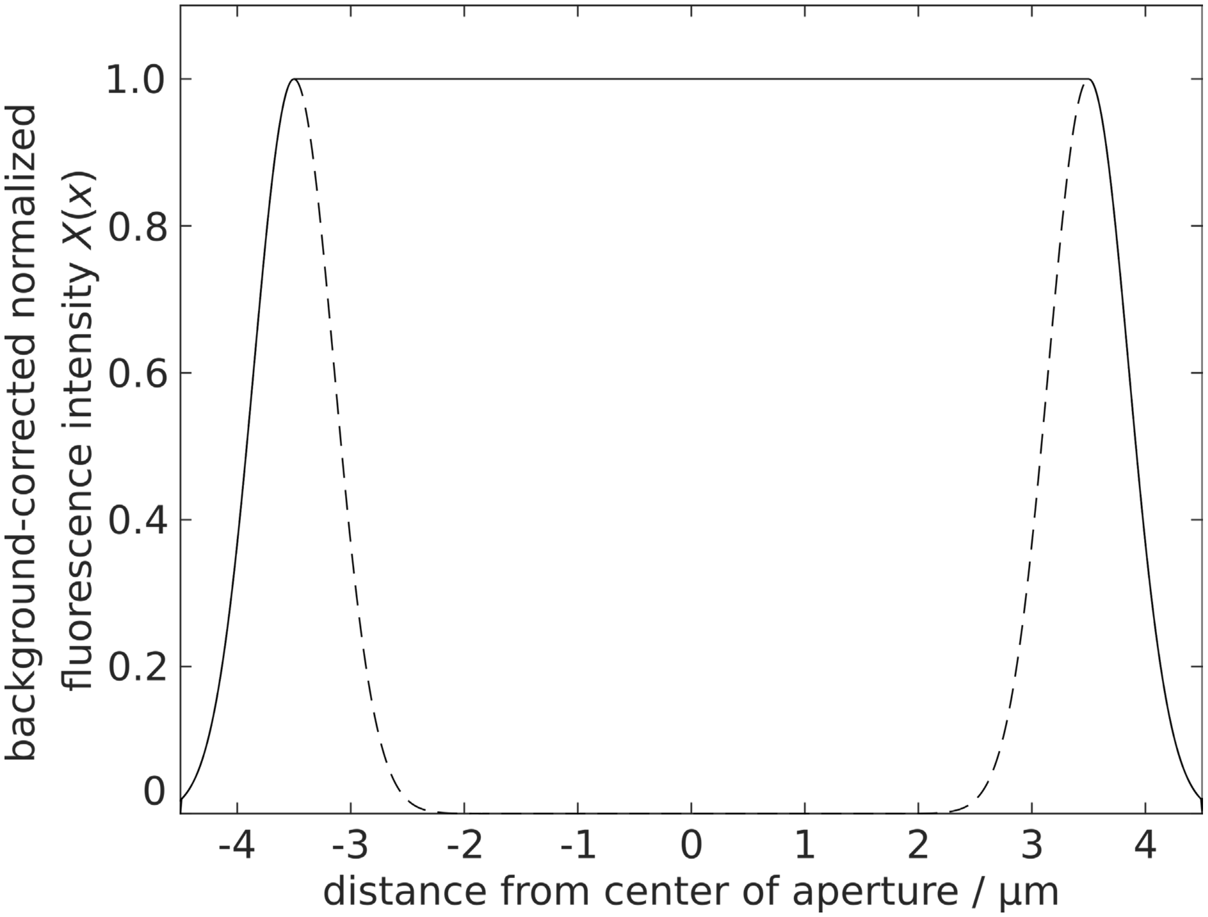

Figure S4: The intensity decay in one dimension was approximated by a Gaussian function with standard deviation $\sigma_{profile}$ = 0.5 µm and the central region by a constant plateau (black solid line). The intensity decay was assumed to reach 0 after d_{edge} = 1 µm. The two-dimensional intensity profile was obtained by multiplying the normalized intensities in *x*- and *y*-direction, $X(x)$ and $Y(y)$, yielding a normalized 2D intensity profile $I(x,y) = X(x) \cdot Y(y)$.

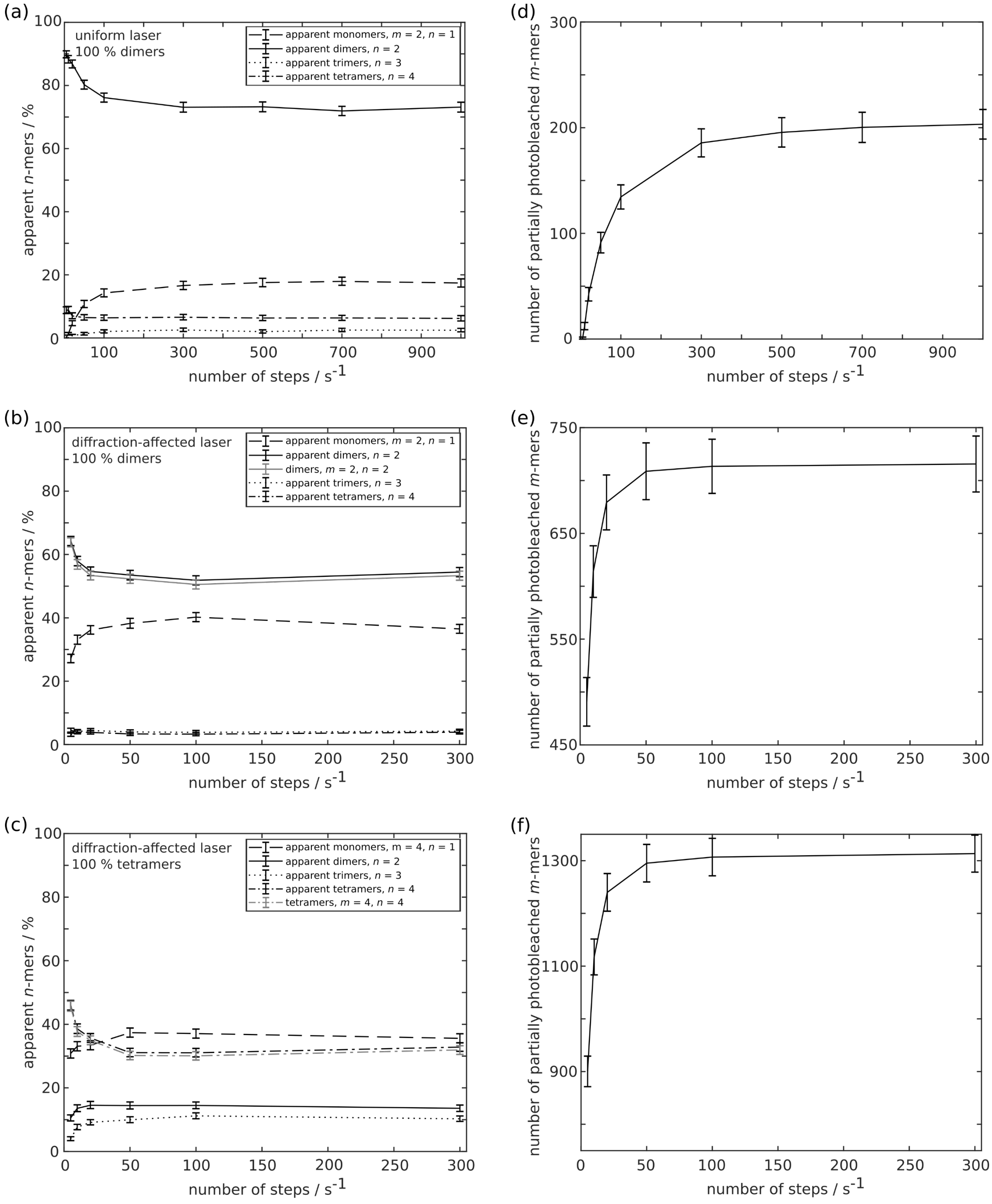

Figure S5 The accuracy of results depends on the time resolution, i.e., on the number of computational steps per simulated time-interval of 1 second. We studied here the convergence of the apparent *n*-mer fractions. A pure dimeric population (a,b,d,e) and a pure tetrameric population (c,f) with $D = 0.5$ µm²/s were exposed to photobleaching for $t_{\mathrm{bleach}} = 0.3$ s (not shown) and $t_{\mathrm{bleach}} = 4$ s assuming a uniform (a,d) or a diffraction-affected (b,c,e,f) laser intensity profile. The optimal t_{rec} and the according analysis region were determined individually for each number of steps. In the uniform case the mean fraction of apparent dimers due to random colocalizations is < 0.2 % and is omitted from the figure. For both photobleaching times, the fraction of apparent tetramers (black dashed-dotted line), the fraction of tetramers (gray dashed-dotted line), the fraction of apparent dimers (black solid line), the fraction of dimers (gray solid line) and the fraction of apparent monomers (black dashed line) do not change substantially for ≥ 300 steps per second in case of the uniform and for ≥ 50 steps per second in case of the diffraction-affected laser intensity profile. The mean number of partially photobleached *m*-mers converges for ~ 500 steps per second in case of the uniform and for ~ 100 steps per second in case of the diffraction-affected laser intensity profile. For all data a minimum of 500 steps (uniform laser intensity profile) or 100 steps (diffraction-affected laser intensity profile) per second were simulated, corresponding to a single time step of 0.002 s (uniform) or 0.01 s (diffraction-affected). Shown are the mean apparent *n*-mer fractions and the error bar (95 % bootstrapping confidence interval) (a-c) and the mean number of partially photobleached *m*-mers and the error bar (simple standard deviation) from 1000 independent simulations (d-f). The lines are a guide to the eye.

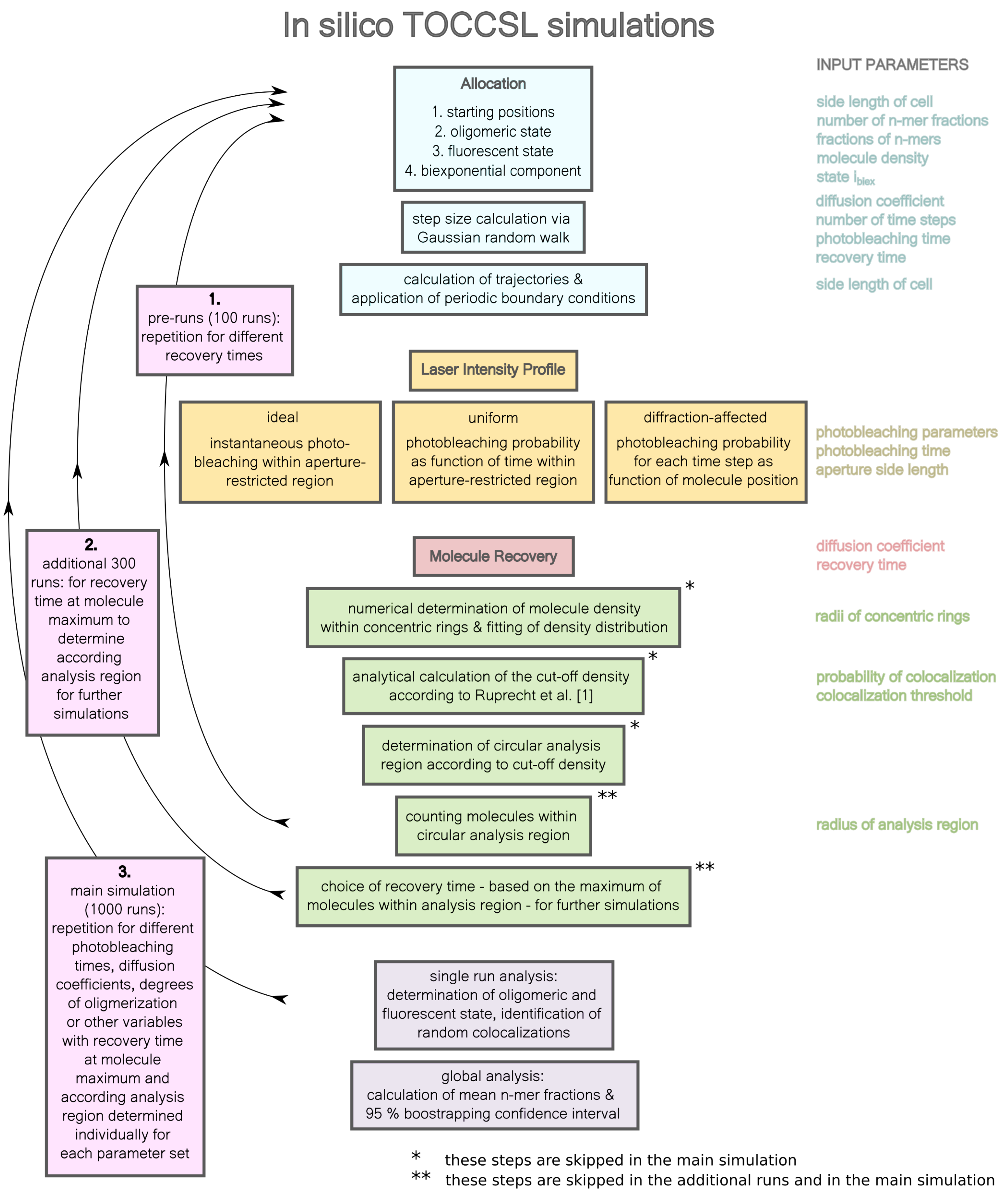

Figure S6 Coordinates x_i and y_i as well as an oligomeric and a fluorescent state are assigned to each molecule for each time step Δt. For each discrete time step Δt individual step sizes for both coordinates, Δx and Δy, are randomly drawn from a one-dimensional Gaussian probability distribution. Additionally, a state i_{biex} is assigned to each oligomeric subunit corresponding to one of two components of a biexponential photobleaching curve. To reproduce the finite size of a cell, periodic boundary conditions are applied. For an ideal, a uniform and a diffraction-affected laser intensity profile, photobleaching is simulated by pre-defining the photobleaching time and the side length of the aperture-restricted region. After photobleaching, molecules re-populate the aperture-restricted region during a pre-set recovery time. After recovery the density distribution within the photobleached area is determined. The analytically determined cut-off density (according to [1]) is compared to the density distribution. The position, at which the local density is equal to the cut-off density is determined and used for calculating the circular analysis region. Recovery is simulated for different recovery times, for each of which the analysis region and the number of molecules within is determined. The recovery time at which the highest number of molecules can be analyzed is used for further simulations, in which other parameters are varied.

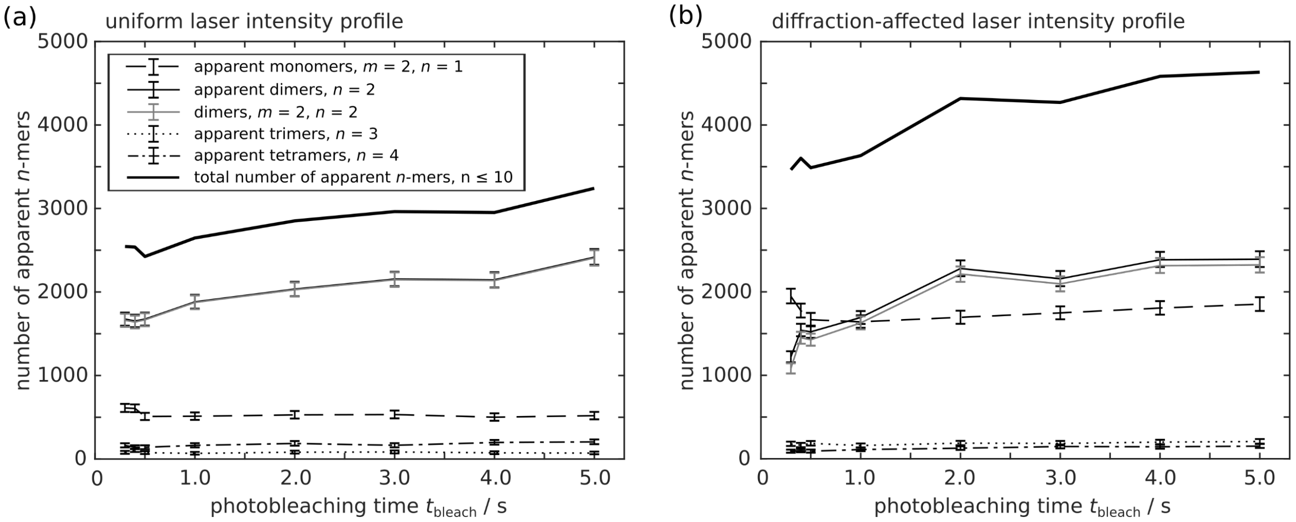

Figure S7 The influence of t_{bleach} on the number of apparent *n*-mers was studied. A purely dimeric population with D = 0.5 µm²/s was exposed to photobleaching assuming a (a) uniform or a (b) diffraction-affected laser intensity profile. According to our standard protocol, we determined for each t_{rec} an analysis region in which the molecule density is below the critical density (Eq. 7), such that the probability of two or more *n*-mers to randomly colocalize is below 20 %. For the t_{rec} at which most molecules are within the analysis region, the number of apparent *n*-mers was counted. This was done independently for different t_{bleach}. As displayed the number of recovering apparent dimers increases with increasing t_{bleach}, which translates to higher fractions of apparent dimers (Fig. 3c). Shown are the number of apparent *n*-mers (sum over 1000 simulation runs) and the error bar (95 % bootstrapping confidence interval) from 1000 independent simulations. The lines are a guide to the eye.

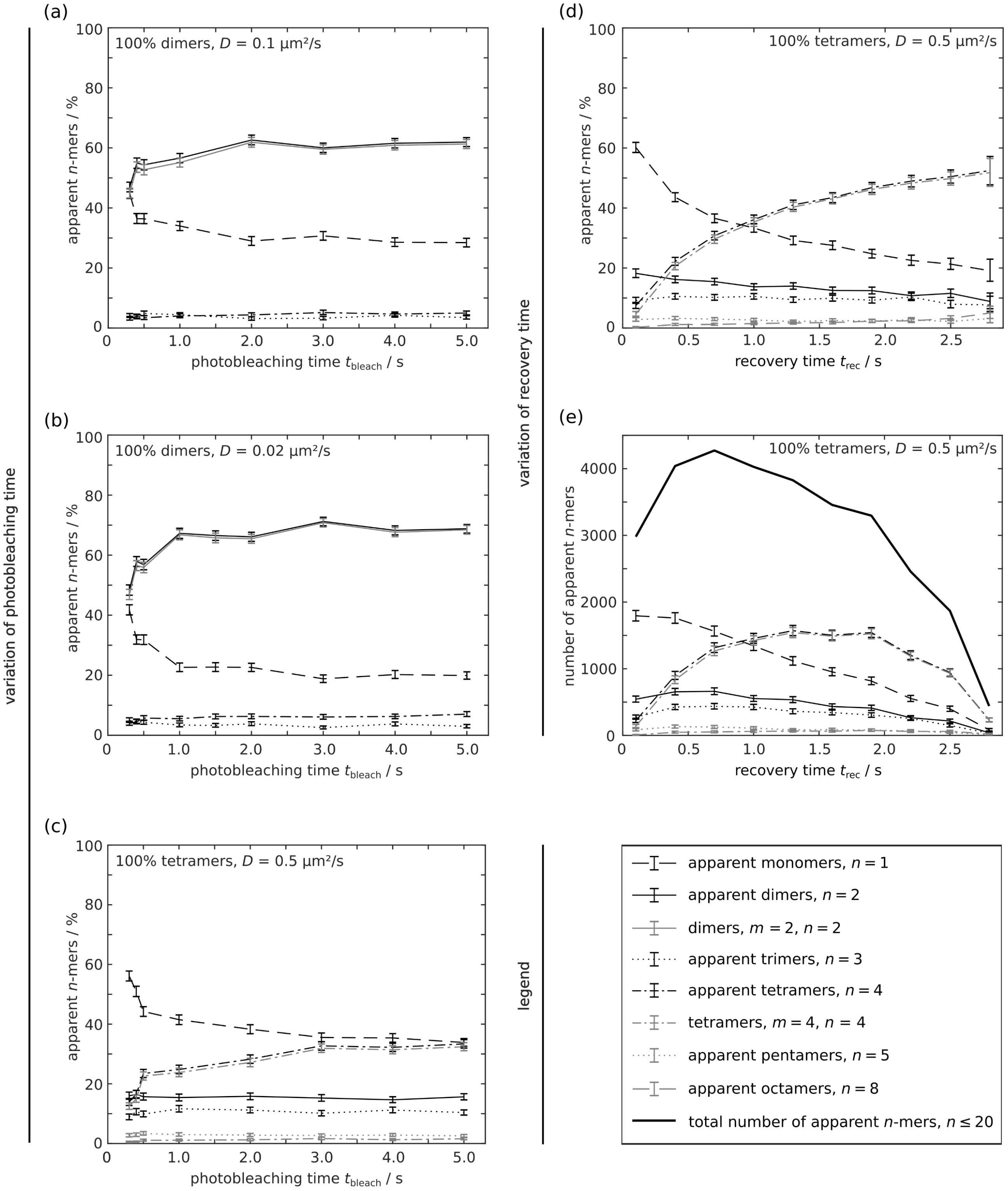

Figure S8 The influence of t_{bleach} (a-c) and t_{rec} (d-e) on the apparent *n*-mer fractions was studied. A purely dimeric population with $D = 0.1$ µm²/s (a) or $D = 0.02$ µm²/s (b) and a purely tetrameric population with $D = 0.5$ µm²/s (c-e) were exposed to photobleaching assuming a diffraction-affected laser intensity profile. The optimal t_{rec} and the according analysis region were determined individually for each t_{bleach}. With increasing t_{rec} the fraction of apparent tetramers increases (d). Shown are the mean apparent *n*-mer fractions (a-d), the number of apparent *n*-mers (sum over 1000 simulation runs) (e) and the error bar (95 % bootstrapping confidence interval) from 1000 independent simulations. The lines are a guide to the eye.

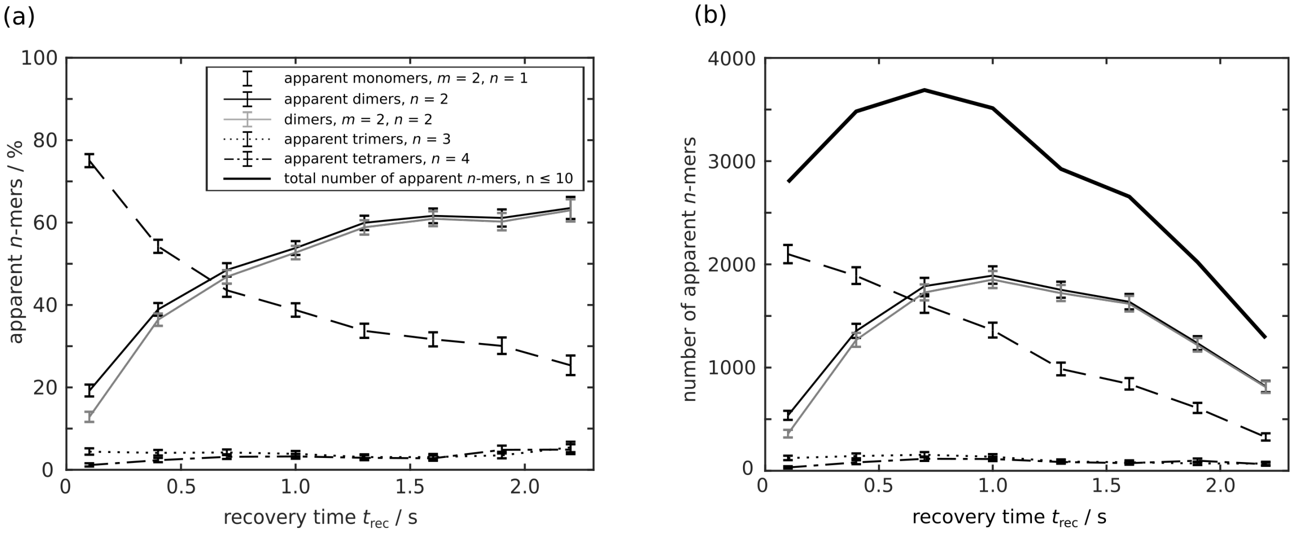

Figure S9 The influence of the shape of the aperture-restricted region was studied. A purely dimeric population with $D = 0.5\ \mu m^2/s$ was exposed to photobleaching assuming a circular aperture with radius $d_{ap} = 7\ \mu m$ and a diffraction-affected laser intensity profile with an intensity decay outside the aperture-restricted region reaching 0 after $d_{edge} = 1\ \mu m$. With increasing t_{rec} the fraction of apparent dimers increases. Shown are the mean apparent n-mer fractions and the error bar (95 % bootstrapping confidence interval) from 1000 independent simulations. The lines are a guide to the eye.

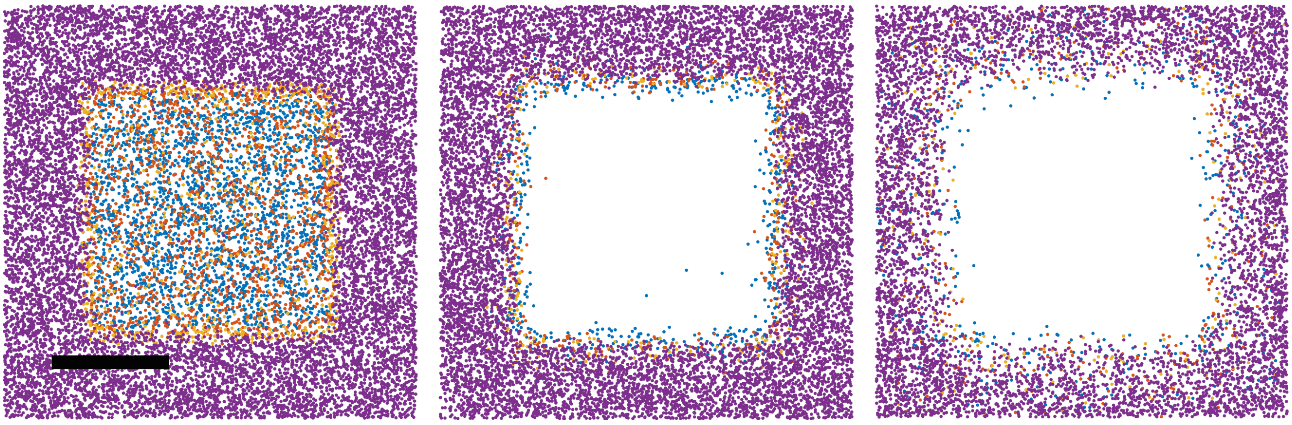

Figure S10 Shown are data from a single simulated TOCCSL experiment, assuming an initial density of 100 tetramers/µm² with a diffusion coefficient of $D = 0.5\ \mu m^2/s$ and diffraction-affected photobleaching. The central aperture-restricted region ($7 \cdot 7\ \mu m^2$) and the additional edge region of 1 µm are photobleached for a single time step ($t_{bleach} = 0.01$ s) (left), for $t_{bleach} = 0.25$ s (middle) and for $t_{bleach} = 4$ s (right). p_{bleach} outside the illuminated area is 0. Tetramers are shown in purple, apparent trimers in yellow, apparent dimers in red and apparent monomers in blue. Scale bar 4 µm.

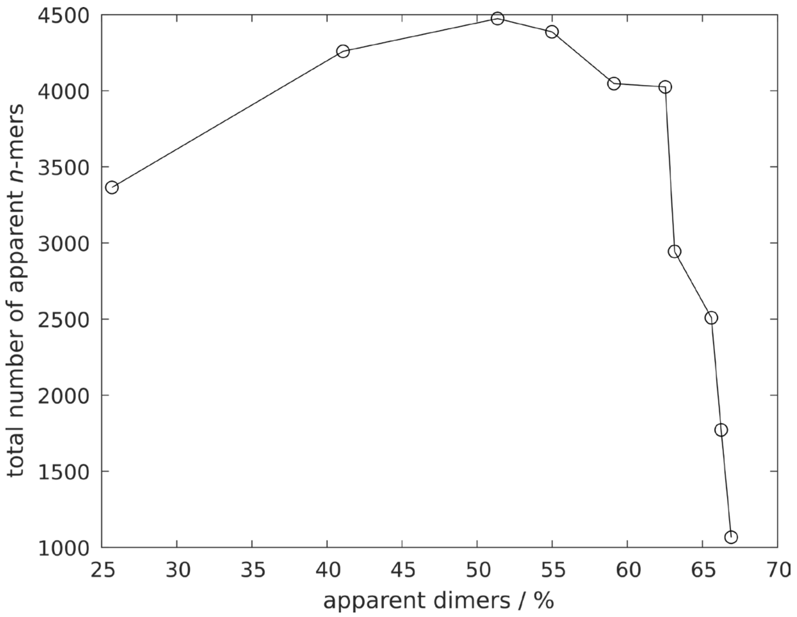

Figure S11. Shown is the total number of apparent *n*-mers within the analysis region plotted against the mean fraction of apparent dimers. A pure dimeric population with $D = 0.5$ µm²/s was exposed to photobleaching for $t_{bleach} = 4$ s, assuming a diffraction-affected laser intensity profile. t_{rec} was varied between 0.1 and 2.8 s and the according analysis region was determined individually for each t_{rec}. With increasing t_{rec} the fraction of apparent dimers increases (Fig. 3d), whereas the sum of apparent *n*-mers first increases with increasing t_{rec} and then decreases for $t_{rec} > 0.7$ s (Fig. 3e). To see the correlation between the apparent dimer fraction and the sum of apparent *n*-mers, data from both Fig. 3d and 3e were combined to generate the presented plot. While at the maximum number of analyzed molecules an apparent dimer fraction of ~ 51 % is determined, the maximal apparent dimer fraction of ~ 67 % is determined from a substantially lower number of analyzed molecules. The lines are a guide to the eye.

Supplemental Tables

Table S1 Fractions of apparent *n*-mers for different photobleaching times assuming a purely dimeric population of molecules and a uniform laser intensity profile.

t_{bleach} / s	apparent monomers	apparent dimers / dimers	apparent trimers	apparent tetramers
0.3	~ 24 %	~ 66 % / ~ 65 %	~ 3 %	~ 6 %
4	~ 17 %	~ 73 % / ~ 72 %	~ 3 %	~ 7 %

Table S2 Fractions of apparent *n*-mers for different photobleaching times assuming a purely dimeric population of molecules and a diffraction-affected laser intensity profile.

t_{bleach} / s	apparent monomers	apparent dimers / dimers	apparent trimers	apparent tetramers
0.3	~ 56 %	~ 35 % / ~ 31 %	~ 5 %	~ 3 %
4	~ 39 %	~ 52 % / ~ 50 %	~ 4 %	~ 3 %

Table S3 Fractions of apparent *n*-mers for different recovery times assuming a purely dimeric population of molecules and a diffraction-affected laser intensity profile.

t_{rec} / s	radius of analysis region / µm	apparent monomers	apparent dimers / dimers	apparent trimers	apparent tetramers
0.1	3.89	~ 63 %	~ 26 % / ~ 18 %	~ 8 %	~ 3 %
0.7	2.98	~ 40 %	~ 51 % / ~ 50 %	~ 4 %	~ 4 %
2.8	0.72	~ 23 %	~ 67 % / ~ 66 %	~ 4 %	~ 6 %

Table S4 Choice of recovery time and analysis region for different diffusion coefficients assuming a purely dimeric population of molecules and a diffraction-affected laser intensity profile.

diffusion coefficient / µm²/s	recovery time / s	radius of analysis region / µm
0.02	16.67	2.85
0.06	5.46	2.93
0.1	3.85	2.8
0.3	1.5	2.71
0.5	0.8	2.87

Table S5 Fraction of apparent *n*-mers for different diffusion coefficients assuming a purely dimeric population of molecules and a diffraction-affected laser intensity profile.

diffusion coefficient / µm² / s	apparent monomers	apparent dimers / dimers	apparent trimers	apparent tetramers
0.02	~ 20 %	~ 68 % / ~ 69 %	~ 4 %	~ 6 %
0.06	~ 28 %	~ 62 % / ~ 61 %	~ 4 %	~ 6 %
0.1	~ 29 %	~ 62 % / ~ 61 %	~ 4 %	~ 5 %
0.3	~34 %	~58 % / ~ 57 %	~ 4 %	~ 3 %
0.5	~39 %	~ 52 % / ~ 50 %	~ 4 %	~ 3 %

Table S6 Fraction of apparent *n*-mers for different diffusion coefficients assuming a 50 % monomeric / 50 % dimeric population of molecules and a diffraction-affected laser intensity profile.

D_2 / D_1	apparent dimers (ideal laser profile)	apparent dimers / dimers (diffraction-affected laser profile)
0.1	~ 10 %	~ 7 % / ~ 0 %
0.2	~ 8 %	~ 8 % / ~ 0 %
0.3	~10 %	~ 9 % / ~ 1 %
0.4	~12 %	~ 10 % / ~ 3 %
0.5	~ 15 %	~ 13 % / ~ 6 %
0.6	~ 23 %	~ 16 % / ~ 10 %
0.7	~ 28 %	~ 22 % / ~ 15 %
0.8	~ 35 %	~ 26 % / ~ 21 %
0.9	~ 40 %	~ 29 % / ~ 25 %
1.0	~ 47 %	~ 33 % / ~ 30 %

Table S7 Fraction of apparent *n*-mers for different diffusion coefficients assuming a purely tetrameric population of molecules and a diffraction-affected laser intensity profile.

diffusion coefficient / µm² / s	apparent monomers	apparent dimers / dimers	apparent trimers / trimers	apparent tetramers / tetramers	apparent pentamers	apparent octamers
0.02	~ 17 %	~ 7 % / ~ 7 %	~ 6 % / ~ 6 %	~ 60 % / ~ 60 %	~ 2 %	~ 4 %
50% 0.02 + 50 % 0.01	~ 22 %	~ 10 % / ~ 10 %	~ 8 % / ~ 7 %	~ 52 % / ~ 52 %	~ 2 %	~ 3 %
0.1	~ 27 %	~ 12 % / ~ 11 %	~ 9 % / ~ 8 %	~ 43 % / ~ 42 %	~ 4 %	~ 2 %
50% 0.1 + 50 % 0.5	~ 28 %	~ 13 % / ~ 13 %	~ 10 % / ~ 10 %	~ 41 % / ~ 41 %	~ 2 %	~ 2 %
0.5	~ 35 %	~ 14 % / ~ 13 %	~ 11 % / ~ 10 %	~ 33 % / ~32 %	~ 3 %	~ 2 %

References

(1) Ruprecht, V., M. Brameshuber & G. J. Schütz. 2010. Two-color single molecule tracking combined with photobleaching for the detection of rare molecular interactions in fluid biomembranes. Soft Matter 6:568-581. doi: 10.1039/B916734J
